# Porcine reproductive and respiratory syndrome virus nonstructural protein 2 promotes the autophagic degradation of adaptor protein SH3KBP1 to antagonize host innate immune responses by enhancing K63-linked polyubiquitination of RIG-I

**DOI:** 10.1371/journal.ppat.1012670

**Published:** 2024-10-28

**Authors:** Jiaoyang Li, Jing Zhang, Pu Sun, Jian Wang, Guoxiu Li, Zhanding Cui, Dong Li, Hong Yuan, Tao Wang, Kun Li, Xingwen Bai, Zhixun Zhao, Yimei Cao, Xueqing Ma, Pinghua Li, Yuanfang Fu, Huifang Bao, Zaixin Liu, Shuqi Xiao, Xinglong Wang, Zengjun Lu

**Affiliations:** 1 State Key Laboratory for Animal Disease Control and Prevention, College of Veterinary Medicine, Lanzhou University, Lanzhou Veterinary Research Institute, Chinese Academy of Agricultural Sciences, Lanzhou, China; 2 College of Veterinary Medicine, Northwest A&F University, Yangling, Shaanxi, China; 3 Gansu Province Research Center for Basic Disciplines of Pathogen Biology, Lanzhou, China; Washington University School of Medicine in Saint Louis: Washington University in St Louis School of Medicine, UNITED STATES OF AMERICA

## Abstract

Non-structural protein 2 (NSP2) of PRRSV is highly variable and plays crucial roles in the virus’s life cycle. To elucidate the function of NSP2 during PRRSV infection, we identified SH3KBP1 as an NSP2-interacting host protein using mass spectrometry. Exogenous SH3KBP1 expression significantly inhibited PRRSV replication by enhancing IFN-I and related ISGs production. Conversely, SH3KBP1 knockdown promoted viral replication by downregulating IFN-I and ISGs levels. *In vivo* experiments revealed that *Sh3kbp1*^*-/-*^ mice were more susceptible to VSV infection, exhibiting reduced serum IFN-β levels. Further investigation showed that SH3KBP1 enhances RIG-I signal transduction by increasing K63-linked polyubiquitination through interaction with the E3 ubiquitin ligase TRIM25. We also found that PRRSV infection and NSP2 overexpression induce the autophagic degradation of SH3KBP1, counteracting the host’s innate immune response. A critical interaction site was identified within the third polyproline-arginine motif in NSP2 (^453^PVPAPR^458^). Recombinant PRRSV lacking this motif displayed reduced virulence and decreased SH3KBP1 degradation. This study advances our understanding of how PRRSV interferes with the host immune response and offers valuable insights for developing novel attenuated vaccines against PRRSV.

## Author summary

PRRS is one of the most devastating diseases affecting the global pig industry. The genome of the PRRS virus (PRRSV) exhibits high variability, characterized by rapid mutation and frequent recombination, particularly in the NSP2 gene. NSP2 plays crucial roles in viral replication, virulence, and immune regulation. This study reveals a novel strategy used by NSP2 to counteract innate immunity. We found that SH3KBP1 enhances the K63-linked polyubiquitination of RIG-I by recruiting TRIM25, thereby stabilizing RIG-I and promoting the production of IFN-I. Mice lacking SH3KBP1 were more susceptible to VSV infection and had lower survival rates. NSP2 directly binds to SH3KBP1 and induces its degradation through the autophagic pathway, aiding viral replication. Additionally, mutations in the amino acid sequences at positions 453–458 within the hypervariable domain of PRRSV NSP2 led to attenuated virulence. This research uncovers a new function for the adaptor protein SH3KBP1 and provides deeper insights into how PRRSV evades host immune responses.

## Introduction

Since its discovery in the 1980s, Porcine Reproductive and Respiratory Syndrome (PRRS) has been an economically devastating viral disease in the global pig industry [1,2]. Porcine reproductive and respiratory syndrome virus (PRRSV), is an enveloped, positive-stranded RNA virus belonging to the order Nidovirales, family Arteriviridae, and the genus Arterivirus [3]. The PRRSV genome is approximately 15.4 kb in length and includes at least 11 open reading frames (ORFs) that encode at least 16 non-structural proteins and 8 structural proteins [4]. Among these, NSP2 is the largest replicase cleavage product and plays a major role in viral genetic variation, replication, virulence, and immune regulation [5].

NSP2 interacts with viral proteins such as NSP3 and NSP5 to form double-layered membrane vesicles that promote PRRSV replication and transcription [6]. Additionally, NSP2TF interacts with GP5 and M, driving PRRSV assembly and budding [7]. NSP2 also interacts with several host proteins to regulate viral replication and the host innate immune response, including recruiting cellular DEAD-box RNA helicase 18 (DDX18) to enhance PRRSV replication, interacting with triggering receptor expressed on myeloid cells 2 (TREM2) to promote PRRSV infection, and counteracting the antiviral function of ISG15 [8–10]. Therefore, identifying host proteins that interact with NSP2 and exploring their mechanism will greatly enhance our understanding of PRRSV pathogenesis.

Innate immunity is the host’s first line of defense against viral infections [11,12]. After virus invasion, pattern recognition receptors recognize the virus and initiate immune responses, ultimately leading to the clearance of the virus by interferons and cytokines [13]. Key pattern recognition receptors include Toll like receptors (TLRs), cyclic GMP AMP synthase (cGAS) like receptors, retinoic acid inducible gene I (RIG-I) like receptors (RLRs), NOD like receptors, and C-type lectin receptors [11] and so on. Among them, the RIG-I signaling pathway is crucial for the host to resist RNA virus infections, such as severe acute respiratory syndrome coronavirus type 2 (SARS CoV-2) and vesicular stomatitis virus (VSV). The K63-linked polyubiquitination of RIG-I is necessary for activating downstream signaling pathways, with TRIM4, TRIM25, and RNF135 being responsible for this modification [14–16]. Research has shown that Zinc-Finger Protein ZCCHC3 recruits TRIM25 to mediate the K63-linked polyubiquitination and activation of RIG-I and MDA5 [17].

Host antiviral proteins activate the host’s natural immune signal and exert antiviral functions, while viral proteins can inhibit antiviral responses by interacting with host antiviral proteins. For example, the influenza A virus NS1 inhibits RIG-I signal transduction by interacting with TRIM25, preventing its dimerization and K63-linked ubiquitination of RIG-I [18]. Similarly, TRIM25 interacts with the N protein of SARS-CoV, thereby inhibiting the activation of RIG-I [19]. The PRRSV N protein inhibits the TRIM25-RIG-I interaction by competitively binding to TRIM25, thereby antagonizing the antiviral activity of TRIM25 [20]. PRRSV NSP1 and NSP2 degrade LGP2 through the ubiquitin-proteasome pathway to evade the host’s innate immune response [21]. In this study, we report that SH3KBP1, a signaling adapter protein, promotes the activation of the RIG-I signaling pathway by enhancing the K63-linked polyubiquitination of RIG-I.

SH3KBP1 (SH3-domain kinase-binding protein 1), also known as Ruk, CIN85, and SETA [22], is a common adaptor protein that is expressed in many tissues [23]. It contains three SH3 domains, a proline-rich region, and a coiled-coil domain [24]. The SH3 domains mediate protein-protein interactions by binding to unique polyproline-arginine motifs. SH3KBP1 is involved in many biological processes, including apoptosis, cell migration [25], positive regulation of B cell activation [26], regulation of cell shape [25], cytoskeleton organization and endocytosis. In the context of viral infections, the absence of the interaction region between Venezuelan equine encephalitis virus (VEEV) NSP3 and SH3KBP1 in the Alphavirus affects the replication of the virus [27]. Mutation in the SH3KBP1 binding motif in the hypervariable domain of chikungunya virus (CHIKV) NSP3 within the same family leads to a weakened viral replication [28]. Moreover, overexpression of SH3KBP1 in HeLa cells inhibits the growth of herpes simplex virus [29]. However, the mechanism by which SH3KBP1 inhibits virus replication and its function in innate immunity remains unclear.

In this study, we found that overexpression of SH3KBP1 inhibited PRRSV replication, while silencing of SH3KBP1 promoted PRRSV replication. During the early stages of viral infection, SH3KBP1 recruited TRIM25 and enhanced the K63-linked polyubiquitination of RIG-I, thereby enhancing the expression IFN-β and ISGs. Conversely, PRRSV and NSP2 reduced the expression of SH3KBP1 through the autophagic pathway; the replication capacity of PRRSV GSWW15-NSP2^Δ453–458^ mutant strain was attenuated. Overall, SH3KBP1 acts as a positive regulator of the anti-PRRSV response. These results provide new insights into the virus-host interaction of mechanisms of PRRSV.

## Results

### SH3KBP1 suppresses PRRSV replication

To identify host proteins that interact with HP-PRRSV NSP2, we cloned the NSP2 gene of the GSWW15 strain into the pCDNA3.1 vector with a 3×Flag tag at its C-terminal. HEK-293T cells were transfected with the plasmid encoding Flag-tagged NSP2, and cell lysates were co-immunoprecipitated with Flag antibody or IgG. The immunoprecipitated proteins were identified and quantified by liquid chromatography-tandem mass spectrometry (LC-MS/MS) analysis. Several host proteins were found to interact with PRRSV NSP2 (Table 1), including previously reported interactors such as CD2AP and HSP70 [5]. Among these proteins, SH3KBP1, which was identified with high confidence by MS with 19 unique peptides against the proteins and 52.14% sequence coverage.

**Table 1:** A list of the top 10 proteins interacting with the PRRSV NSP2 protein.

Protein ID	Protein Qscore	Protein Mass	Unique Peptide Num	Description
sp|Q9Y5K6	94.06	71407.32	25	CD2AP (CD2-associated protein)
sp|Q96B97	72.19	73081.70	19	SH3KBP1 (SH3 domain-containing kinase-binding protein 1)
sp|P52907	35.07	32902.33	9	CAZA1 (F-actin-capping protein subunit alpha-1)
sp|Q13363	22.06	47505.49	6	CTBP1 (C-terminal-binding protein 1)
sp|P47755	20.64	32928.61	5	CAZA2 (F-actin-capping protein subunit alpha-2)
sp|P56545	18.67	48914.11	5	CTBP2 (C-terminal-binding protein 2)
sp|P12236	12.46	32845.19	4	ADT3 (ADP/ATP translocase 3)
sp|P35580	12.39	228857.95	3	MYH10 (Myosin-10)
sp|P11940	9.40	70625.87	3	PABP1 (Polyadenylate-binding protein 1)
sp|P01023	9.09	163187.889	3	A2MG (Alpha-2-macroglobulin)

**Fig 1:**
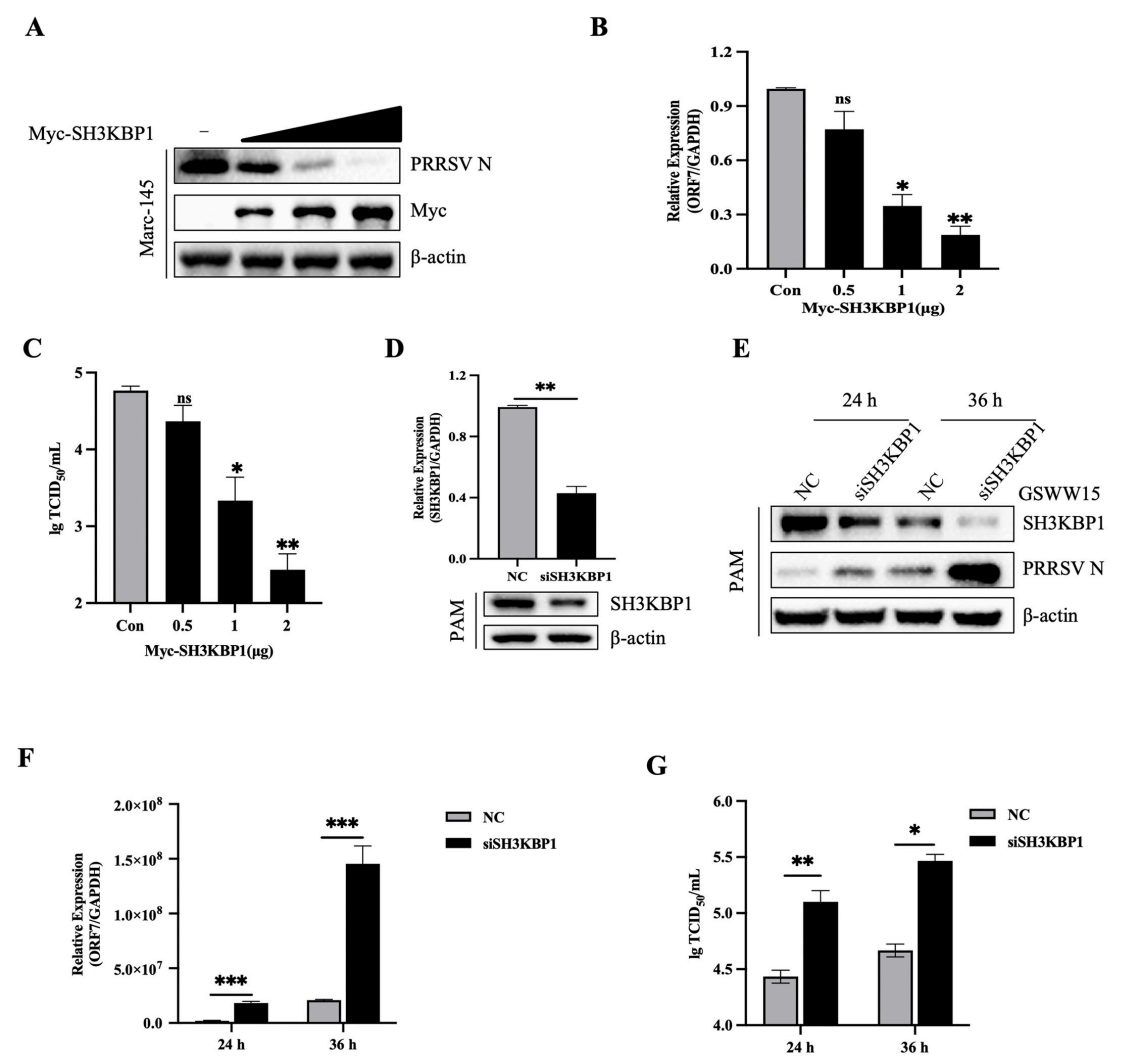
SH3KBP1 suppresses PRRSV replication. **(A-C)** Marc-145 cells were transfected with Myc-EV or different doses of Myc-SH3KBP1, and then infected with PRRSV (MOI = 0.1) for 36 h. The cell lysates and culture supernatants were collected to analyze PRRSV N protein expression and viral titers with Western blot (A), RT-qPCR (B), and TCID_50_ assay (C). **(D)** PAM cells were transfected with NC or siSH3KBP1 for 36 h, RT-qPCR (top) and Western blot (bottom) were used to analyze the expression level of SH3KBP1 in the cells, respectively. **(E-G)** PAM cells were transfected with NC or siSH3KBP1 for 36 h and then infected with PRRSV (MOI = 0.1) for different time periods. The cells were harvested for Western blot (E), RT-qPCR (F) and TCID_50_ assay (G). Data are representative of three independent experiments. Data are expressed as mean ± SD replicates of three independent experiments (*P < 0.05, **P < 0.01, ***P < 0.001; unpaired, two-tailed Student’s *t* test).

To investigate the role of SH3KBP1 in PRRSV replication, Marc-145 cells were transfected with empty vector or different doses of SH3KBP1-expressing plasmids. At 24 hours (h) post-transfection, the cells were infected with PRRSV (MOI = 0.1), and virus replication was assessed at 24 hours post-infection (hpi) via qPCR and Western blot. The results showed significant reductions in viral RNA and protein levels in SH3KBP1-overexpressing cells compared to control cells (Fig 1A and 1B). Furthermore, viral titers in the supernatant of SH3KBP1-overexpressing cell were significantly reduced (~2 logs) (Fig 1C). Indirect immunofluorescence (IFA) results confirmed decreased expression of PRRSV N protein in SH3KBP1-overexpressing cells (S1A Fig). Conversely, SH3KBP1 knockdown in PAMs via siRNAs resulted in increased viral titers, protein levels, and transcription levels (Fig 1D–1G). IFA results showed significantly higher fluorescence intensity of PRRSV N protein in SH3KBP1 knockdown in Marc-145 cells compared to control cells (S1B Fig). To test whether SH3KBP1 affects cell viability, we performed CCK-8 assays after SH3KBP1 overexpression or knockdown. The results showed that neither overexpression nor knockdown of SH3KBP1 affects cell viability (S1C and S1D Fig). These results indicated that downregulation of SH3KBP1 significantly enhances PRRSV replication.

### SH3KBP1 promotes IFN-β production and ISG expression *in vitro* and *in vivo*

**Fig 2:**
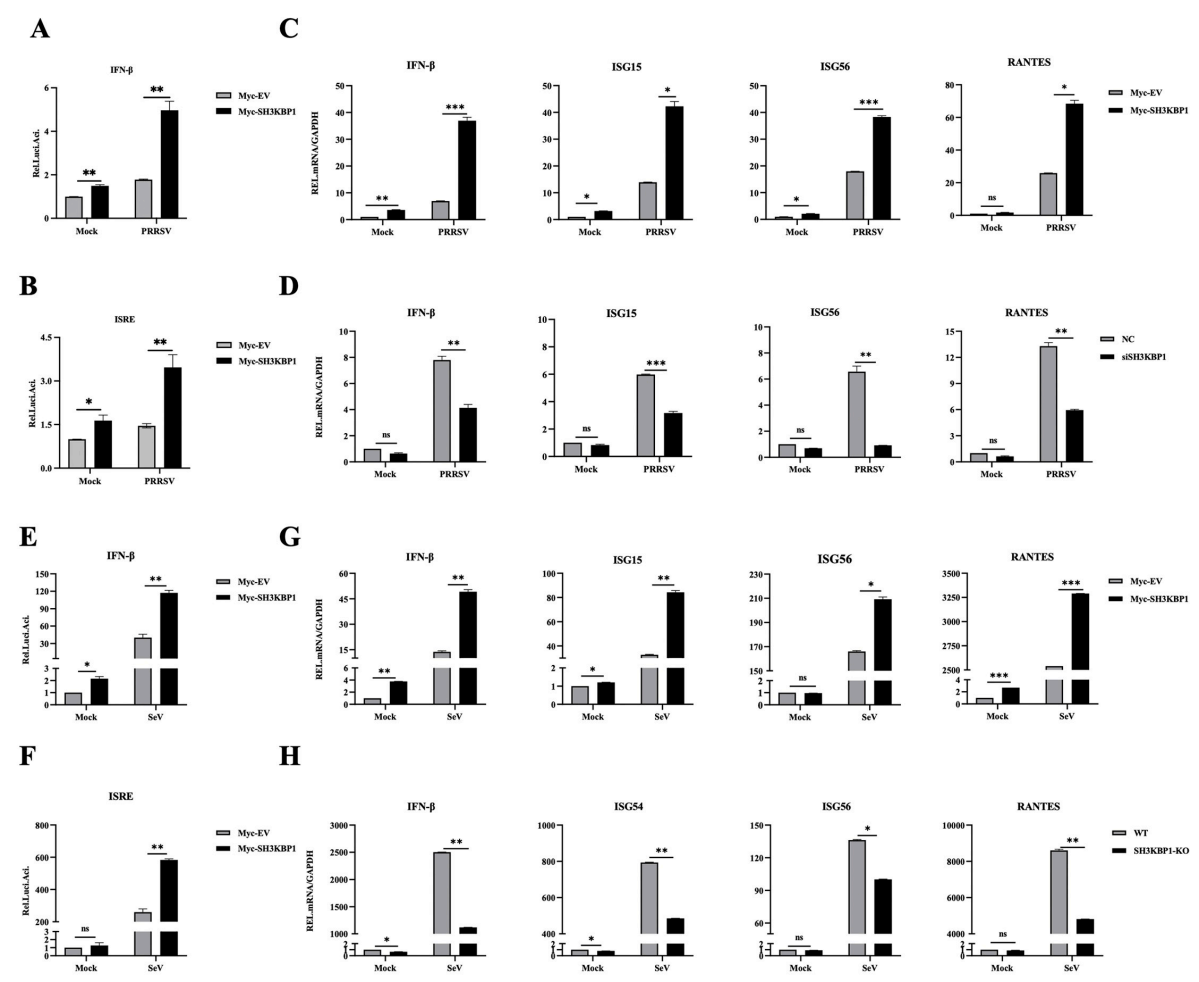
SH3KBP1 promotes IFN-β production and ISGs expression. **(A-B)** Luciferase reporter assays of IFN-β or ISRE promoter activity of Marc-145 cells that were co-transfected with the indicated reporter, pRL-TK, and control Myc-EV or Myc-SH3KBP1 for 24 h followed by infection with PRRSV for 36 h. **(C)** RT-qPCR analysis was performed to measure IFN-β, ISG15, ISG56, and RANTES mRNA levels in Marc-145 cells transfected with Myc-EV or Myc-SH3KBP1, followed by infection with PRRSV (MOI = 0.1) at the indicated time points. **(D)** RT-qPCR analysis of IFN-β, ISG15, ISG56 and RANTES mRNA in Marc-145 cells transfected with control siRNA (NC) or siSH3KBP1 targeting SH3KBP1 for 36 h followed by infection with PRRSV for 36 h. **(E-F)** Luciferase reporter assays of IFN-β or ISRE promoter activity of HEK-293T cells that were co-transfected with the indicated reporter, pRL-TK, and control Myc-EV or Myc-SH3KBP1 for 24 h followed by infection with SeV for 12 h. **(G)** RT-qPCR analysis of IFN-β, ISG15, ISG56 and RANTES mRNA levels in HEK-293T cells transfected with Myc-EV or Myc-SH3KBP1 followed by infection with SeV (MOI = 1) at indicated time points. **(H)** RT-qPCR detection of IFN-β, ISG54, ISG56 and RANTES mRNA levels in WT and SH3KBP1-KO HEK-293T cells infected with SeV (MOI = 1) for 12 h. Data of are expressed as mean ± SD replicates of three independent experiments (*P < 0.05, **P < 0.01, ***P < 0.001; unpaired, two-tailed Student’s *t* test).

To explore how SH3KBP1 restrains PRRSV replication, we examined its role in IFN signaling, a crucial aspect of host antiviral immunity. Viral infection initiates signaling cascades for the production of IFNs-I and the expression of various ISGs. Dual luciferase report assay demonstrated that SH3KBP1 activates IFN-β and ISRE promoters (Fig 2A and 2B). qPCR results showed that SH3KBP1 overexpression in Marc-145 cells effectively activated IFN-β and ISGs such as ISG15, ISG56 and RANTES upon PRRSV infection (Fig 2C).

Conversely, SH3KBP1 knockdown by siRNA in Marc-145 cells resulted in downregulated expression of these genes upon PRRSV infection (Fig 2D). In HEK-293T cells, luciferase reporter assays demonstrated that SH3KBP1 overexpression increase basal and SeV-induced activation of IFN-β and ISRE promoters (Fig 2E and 2F). qPCR analysis confirmed upregulated expression of IFN-β, ISG15, ISG56 and RANTES in SH3KBP1-overexpressing cells upon SeV infection (Fig 2G), while SH3KBP1 knockout (KO) cells exhibited significantly downregulated expression of these genes upon SeV infection (Fig 2H). Moreover, Western blot and ELISA results confirmed that upon SeV infection, the protein levels of IFN-β and ISGs were increased in SH3KBP1-overexpressing HEK-293T cells and decreased in SH3KBP1-KO cells (S2A–S2D Fig).

**Fig 3:**
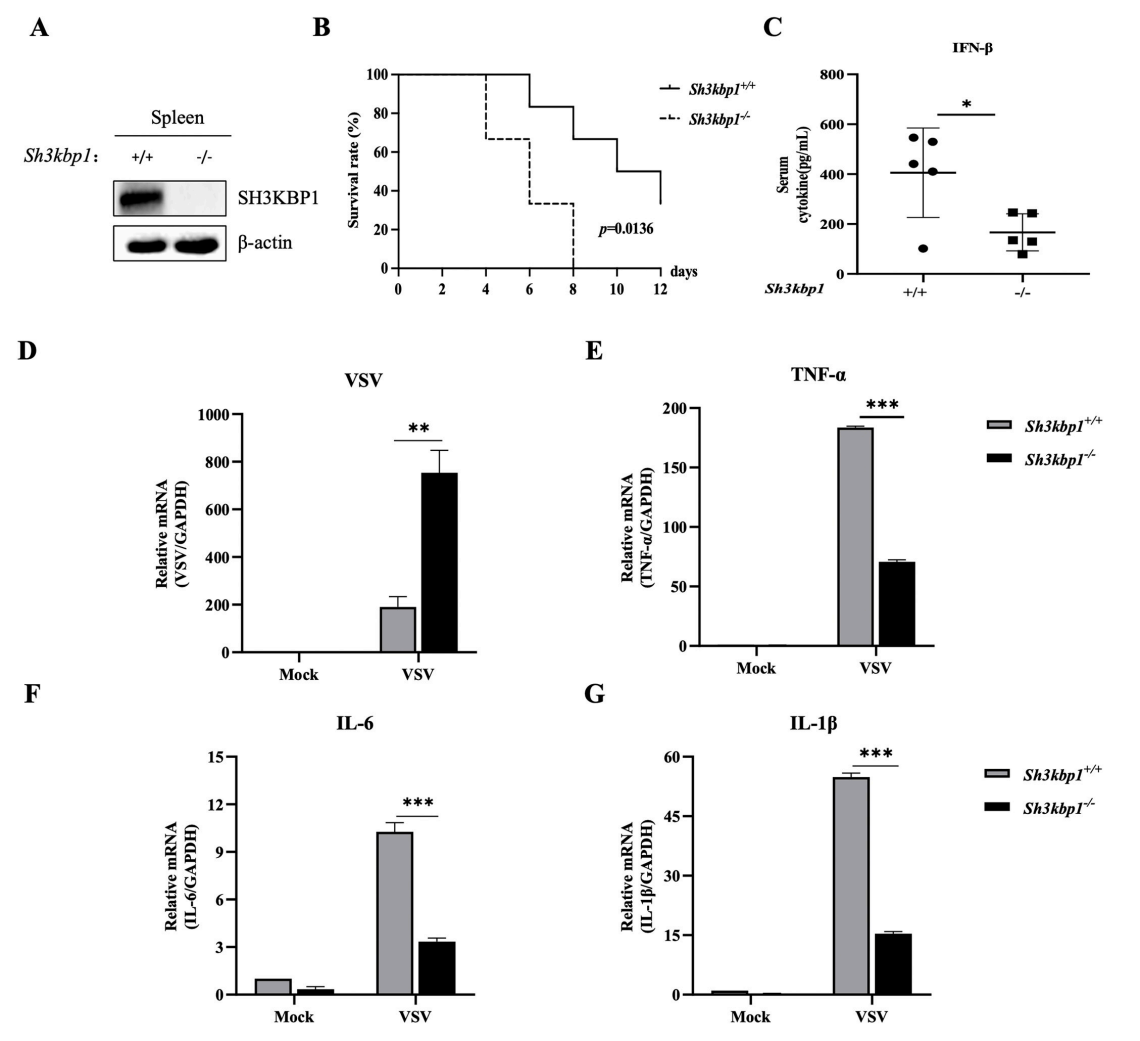
SH3KBP1 enhanced the innate antiviral response *in vivo*. **(A)** Deficiency of *Sh3kbp1* in the mice was confirmed by Western blot. **(B)** Sex- and age-matched *Sh3kbp1*^+/+^ and *Sh3kbp1*^-/-^ mice (n = 6 for each group) were tail intravenous injected with VSV (1×10^8^ PFU per mouse), and mouse survival rate was recorded daily for 12 days. **(C)** *Sh3kbp1*^+/+^ and *Sh3kbp1*^-/-^ mice (n = 5 for each group) were tail intravenous injected with VSV (1×10^8^ PFU per mouse), 6 h later, IFN-β production in the serum were measured by ELISA. **(D-G)** *Sh3kbp1*^+/+^ and *Sh3kbp1*^-/-^ mice (n = 3 for each group) were tail intravenous injected with VSV (1×10^8^ PFU per mouse) for 4 days. The mRNA levels of VSV, TNF-α, IL-6 and IL-1β in the spleens of mice were quantified by RT-qPCR. Data are expressed as mean ±SD replicates of three independent experiments (*P<0.05, **P<0.01, ***P<0.001; unpaired, two-tailed Student’s *t* test).

To verify whether SH3KBP1 promotes IFN production *in vivo*, we generated SH3KBP1 knockout (KO) mice lacking exon2 using the CRISPR/Cas9 system. SH3KBP1 expression was efficiently suppressed in *Sh3kbp1*^-/-^ mice (Fig 3A). We then challenged *Sh3kbp1*^-/-^ mice with VSV via tail intravenous injection. The survival rate of *Sh3kbp1*^-/-^ mice was lower compared to *Sh3kbp1*^+/+^ mice (Fig 3B). Meanwhile, IFN-β levels in the serum of *Sh3kbp1*^-/-^ mice were significantly lower after VSV infection at 6 h compared to *Sh3kbp1*^+/+^ mice (Fig 3C). Correspondingly, VSV replication was significantly increased in the spleen of *Sh3kbp1*^-/-^ mice compared to *Sh3kbp1*^+/+^ mice (Fig 3D). In addition, the mRNA levels of TNF-α, IL-6, and IL-1β in the spleen of *Sh3kbp1*^-/-^ mice were significantly lower than in *Sh3kbp1*^+/+^ mice 96 hpi (Fig 3E–3G). These data showed that *Sh3kbp1*^-/-^ mice were more susceptible to VSV infection by impairing the production of IFNs and inflammatory cytokines, further demonstrated that SH3KBP1 significantly enhanced the innate antiviral response.

### SH3KBP1 enhances the expression of RIG-I

**Fig 4:**
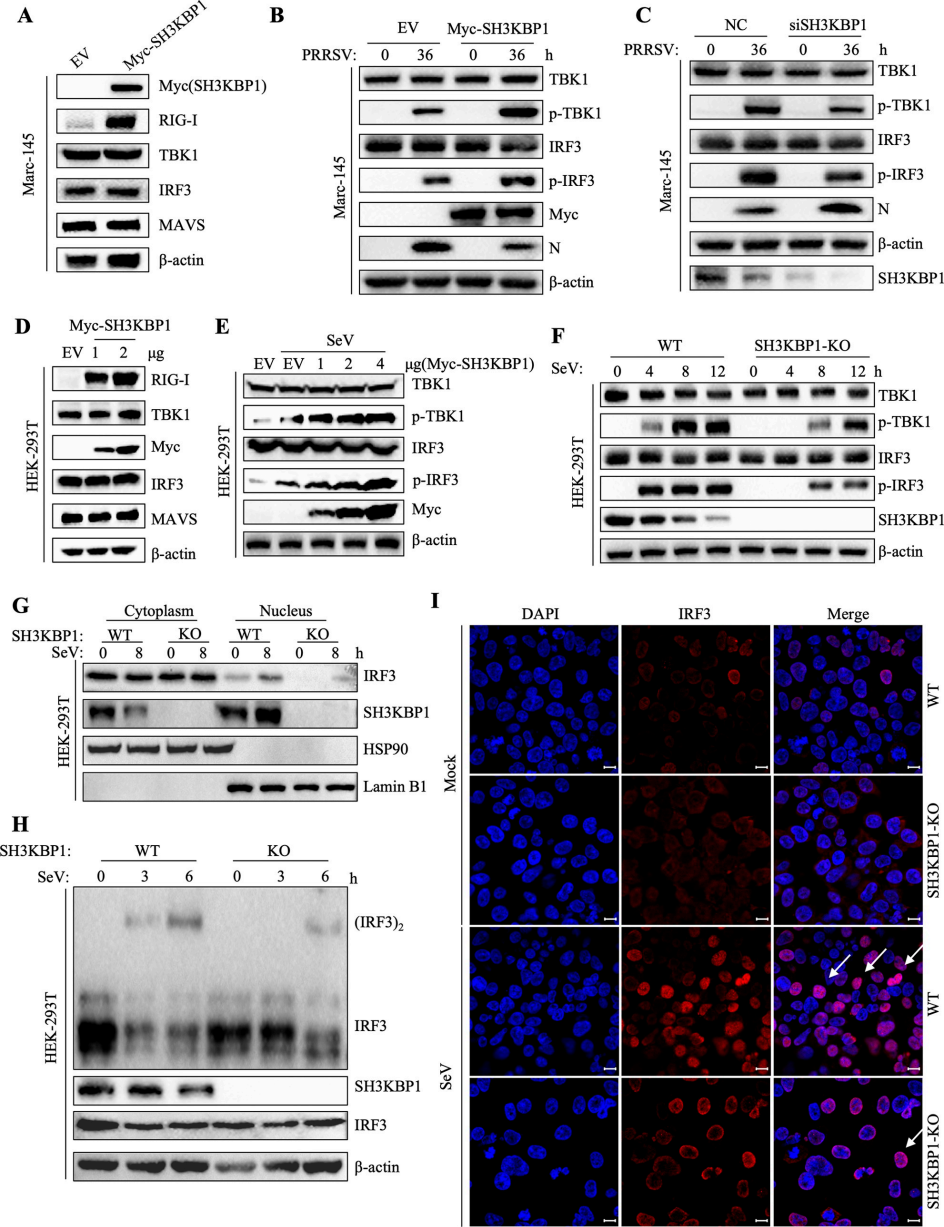
SH3KBP1 increased the protein level of endogenous RIG-I. **(A)** Marc-145 cells were transfected with Myc-EV or Myc-SH3KBP1 and then Western blot analysis was performed with the specified antibodies. **(B)** Marc-145 cells were transfected with the Myc-SH3KBP1 or Myc-EV for 24 h and inoculated with PRRSV (MOI = 0.1). The cell lysates were then subjected to Western blot analysis with the specified antibody. **(C)** Marc-145 cells were transfected with the NC or siSH3KBP1 for 36 h and inoculated with PRRSV (MOI = 0.1). Cell lysates were then subjected to Western blot with the indicated antibodies. **(D)** HEK-293T cells were transfected with different doses of Myc-SH3KBP1 or Myc-EV, and the protein levels of immune adaptor proteins were detected by Western blot. **(E)** HEK-293T cells were transfected with different doses of Myc-SH3KBP1 for 24 h, transfected cells were mock infected or infected with SeV, samples were collected 8 h after infection, and the levels of phosphorylated TBK1 and phosphorylated IRF3 were detected by Western blot. **(F)** WT and SH3KBP1-KO cell lines were mock infected or infected with SeV. Samples were collected at different time points after infection, and the levels of phosphorylated TBK1 and phosphorylated IRF3 were detected by Western blot. **(G)** Western blot of cytoplasmic (Cyto) and nuclear extracts from WT and SH3KBP1-KO HEK-293T cells that were mock infected or infected with SeV for 8 h. The cytoplasmic and nuclear extracts were analyzed by Western blot with antibodies against the indicated proteins. **(H)** WT and SH3KBP1-KO HEK-293T cells were mock infected or infected with SeV for the indicated times before native PAGE and SDS PAGE analyses. **(I)** WT and SH3KBP1-KO HEK-293T cells were mock infected or infected with SeV for the indicated times, the cells were fixed and subjected to confocal imaging to detect the IRF3 distribution. Scale bars, 10 μm. Data are representative of three independent experiments.

Since RIG-I is the key pathogen recognition receptor activated upon PRRSV infection [30], we investigated whether SH3KBP1 enhances IFN production through regulating RLR signaling. Overexpression of Myc-SH3KBP1 in Marc-145 cells significantly increased RIG-I protein levels, without affecting TBK1, MAVS, and IRF3 protein levels (Fig 4A). We also confirmed that in iPAM cells, overexpression of SH3KBP1 elevated RIG-I protein level (S3A Fig). Additionally, we detected the mRNA level of RIG-I in SH3KBP1 overexpression cells. The results showed that SH3KBP1 did not induce an increase in RIG-I transcription level (S3B Fig). PRRSV infection in SH3KBP1-overexpressing Marc-145 cells resulted in upregulated phosphorylation of TBK1 and IRF3 (Fig 4B), while SH3KBP1 knockdown decreased their phosphorylation (Fig 4C). In HEK-293T cells, SH3KBP1 overexpression increased RIG-I protein levels in a dose dependent manner (Fig 4D). As SH3KBP1 expression increased, the levels of phosphorylated TBK1 and IRF3 were significantly elevated following SeV infection in HEK-293T cells (Fig 4E). Conversely, SH3KBP1-KO cells showed reduced TBK1 and IRF3 phosphorylation compared to wild-type (WT) cells after SeV infection (Fig 4F). Additionally, we performed nuclear-cytoplasmic fractionation, and the Western blot results showed that SeV infection-induced IRF3 nuclear translocation was inhibited in SH3KBP1 knockout cells (Fig 4G). Consistent with the nuclear-cytoplasmic fractionation results, SeV- induced dimerization of IRF3 was affected by knockout of SH3KBP1 in HEK-293T cells (Fig 4H). Confocal microscopy results showed that after SeV infection, nuclear translocation of IRF3 was reduced in SH3KBP1-KO cells compared to WT cells (Fig 4I), indicating that SH3KBP1 facilitates IRF3 activation. We also performed a CCK-8 assay to ensure that knocking out SH3KBP1 did not affect cell viability (S3C Fig). These findings suggest that SH3KBP1 positively regulated the expression of IFN-β by up-regulating the expression of RIG-I, thereby impairing viral replication.

### SH3KBP1 promotes K63-Linked polyubiquitination of RIG-I by recruiting TRIM25

**Fig 5:**
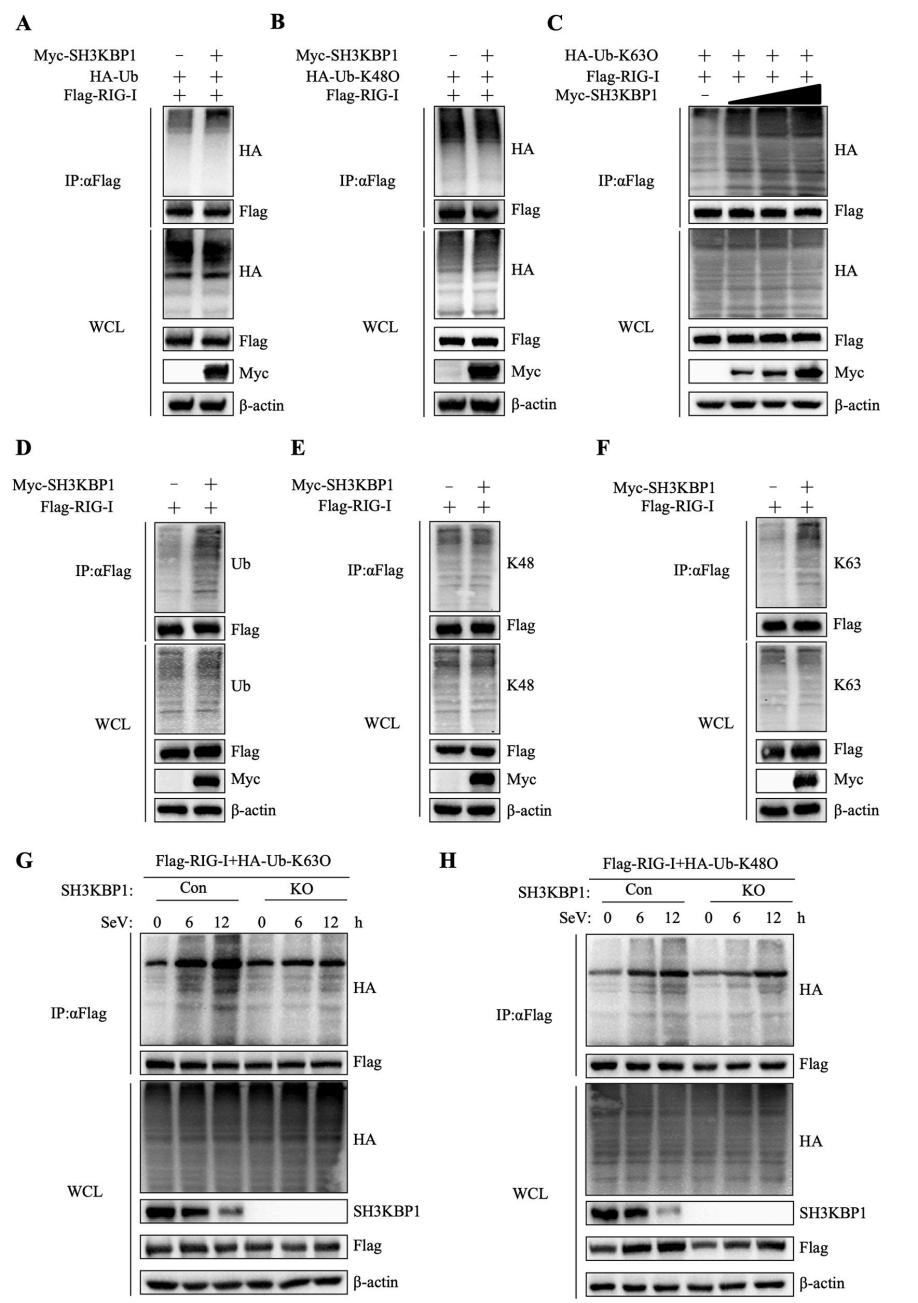
SH3KBP1 enhances K63-linked polyubiquitination of RIG-I. **(A-B)** HEK-293T cells were transfected with Flag-RIG-I, HA-Ub or HA-K48O together with Myc-EV or Myc-SH3KBP1. Cell lysates were subjected to Co-IP with anti-Flag antibody followed by Western blot analysis with indicated antibodies. **(C)** HEK-293T cells were transfected with Flag-RIG-I and HA-K63O together with a gradient dose of Myc-SH3KBP1 for 24 h, followed by IP and Western blot analysis with the indicated antibodies. **(D-F)** HEK-293T cells were co-transfected with Flag-RIG-I, HA-K63O, and Myc-EV or Myc-SH3KBP1 for 24 h. Subsequently, immunoprecipitation and Western blot analyses were performed using the indicated antibodies. **(G-H)** WT and SH3KBP1-KO HEK-293T cells were transfected with Flag-RIG-I and HA-Ub (K63O/K48O) for 24 h, then infected with SeV for the indicated time points before Co-IP and Western blot analysis with the indicated antibodies. Data are representative of three independent experiments.

Post-translational modifications, particularly ubiquitination, are crucial for regulating RIG-I stability. We investigated the involved of SH3KBP1 in this process. In HEK-293T cells, we co-transfected Flag-tagged RIG-I, Myc-tagged SH3KBP1, and HA-tagged K63- or K48 ubiquitin. Co-immunoprecipitation (Co-IP) revealed that overexpression of SH3KBP1 significantly increased RIG-I polyubiquitination (Fig 5A). However, overexpression of SH3KBP1 did not affect K48-linked ubiquitination (Fig 5B). Subsequently, we investigated its impact on K63-linked polyubiquitination. In vivo ubiquitination assays demonstrated that increasing SH3KBP1 expression enhanced K63-linked polyubiquitination of RIG-I (Fig 5C). We transfected Myc-tagged SH3KBP1 and Flag- RIG-I into HEK-293T cells. Cell lysates were immunoprecipitated using a Flag antibody and then blotted for Ubiquitin, K48-Ubiquitin, and K63-Ubiquitin. The results further confirmed that SH3KBP1 promotes K63-linked polyubiquitination of RIG-I but not K48-linked polyubiquitination (Fig 5D–5F). To investigate the effect of SH3KBP1 deficiency on the ubiquitination level of RIG-I, Flag-tagged RIG-I and HA-tagged K63 or K48 ubiquitin were co-transfected into WT or SH3KBP1-KO cells. The cells were then infected with SeV, and the ubiquitination levels of RIG-I were detected at different time points post-infection. The results showed that SH3KBP1 deficiency significantly reduced the K63-linked polyubiquitination of RIG-I induced by SeV infection (Fig 5G) but did not affect its K48-linked polyubiquitination (Fig 5H). To explore the mechanism that SH3KBP1 enhance RIG-I polyubiquitination, we detected whether RIG-I interacted with SH3KBP1. To our surprise, Co-IP results showed that SH3KBP1 did not interact with RIG-I (S4A Fig). Therefore, the above data suggest that SH3KBP1 is a positive regulator of RIG-I stability, but does not have a direct interaction with RIG-I.

**Fig 6:**
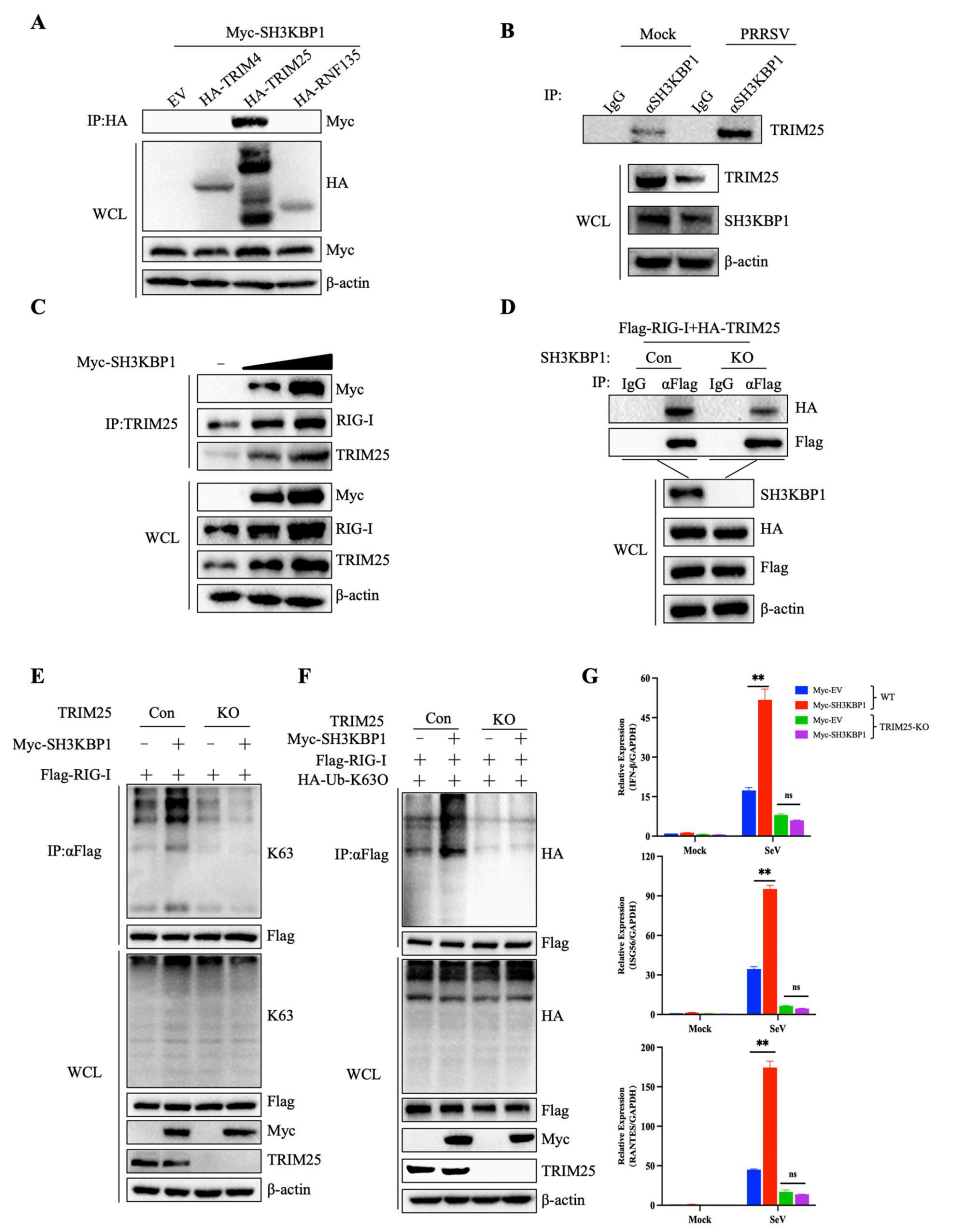
SH3KBP1 promotes K63-linked polyubiquitination of RIG-I by recruiting TRIM25. **(A)** HEK-293T cells were transfected with the indicated plasmids for 24 h. Co-IP and Western blot analyses were performed with indicated antibodies. **(B)** Marc-145 cells were mock infected or infected with PRRSV for the indicated times. Immunoprecipitation was performed with SH3KBP1 antibody. Western blot analysis was performed with indicated antibodies. **(C)** HEK-293T cells were transfected with Flag-RIG-I and HA-TRIM25, along with Myc-EV or varying doses of Myc-SH3KBP1 plasmid for 24 h. Subsequently, Co-IP and Western blot analysis were performed using the indicated antibodies. **(D)** WT and SH3KBP1-KO HEK-293T cells were transfected with Flag-RIG-I and HA-TRIM25 for 24 h. Following transfection, Co-IP and Western blot analysis were conducted using the indicated antibodies. **(E)** WT and TRIM25-KO HEK-293 cells were transfected with Flag-RIG-I, Myc-EV or Myc-SH3KBP1 for 24 h, followed by Co-IP and Western blot analysis with the indicated antibodies. **(F)** WT and TRIM25-KO HEK-293 cells were transfected with Flag-RIG-I, HA-K63O, Myc-EV or Myc-SH3KBP1 for 24 h, followed by Co-IP and Western blot analysis with the indicated antibodies. **(G)** RT-qPCR assay of I IFN-β, ISG56 and RANTES expression in WT and TRIM25-KO HEK-293 cells with SeV (MOI = 1) infected for 12 h after transfection with Myc-EV or Myc-SH3KBP1. Data are representative of three independent experiments. Data are expressed as mean ± SD replicates of three independent experiments (*P < 0.05, **P < 0.01, ***P < 0.001; unpaired, two-tailed Student’s *t* test).

Three E3 ubiquitin ligases, including TRIM25, TRIM4 and RNF135, have been identified mediating K63-linked polyubiquitination of RIG-I [14,16,31]. Co-IP results showed that SH3KBP1 specifically interacted with TRIM25 but did not interact with TRIM4 and RNF135 (Fig 6A). In uninfected cells, endogenous SH3KBP1 interacted with TRIM25, and this interaction was significantly enhanced after viral infection (Fig 6B). Furthermore, Co-IP results showed that overexpression of SH3KBP1 promoted the binding of TRIM25 with RIG-I (Fig 6C), while SH3KBP1 deficiency weakened their binding (Fig 6D). To further verify the role of endogenous TRIM25 in the interaction between SH3KBP1 and RIG-I, we used a TRIM25-KO cell line and assessed its cellular activity (S4B Fig). In addition, Co-IP experiments showed that overexpression of SH3KBP1 did not enhance the K63-linked polyubiquitination of RIG-I in TRIM25-deficient cells (Fig 6E and 6F). Similarly, SH3KBP1-induced mRNA and protein levels of IFN-β, ISG56, and other ISGs was abolished by TRIM25 deficiency (Figs 6G and S4C–S4D). To further explore the interaction domain of SH3KBP1 and TRIM25, we constructed truncations of both proteins and performed Co-IP with these mutants. The results showed that SH3KBP1 interacts with TRIM25 through its SH3-ABC fragment, while TRIM25 interacts with SH3KBP1 through its BB/CCD domain (S4E and S4F Fig). These results indicated that SH3KBP1 enhanced TRIM25-mediated K63-linked polyubiquitination of RIG-I.

### PRRSV replication induced SH3KBP1 degradation

**Fig 7:**
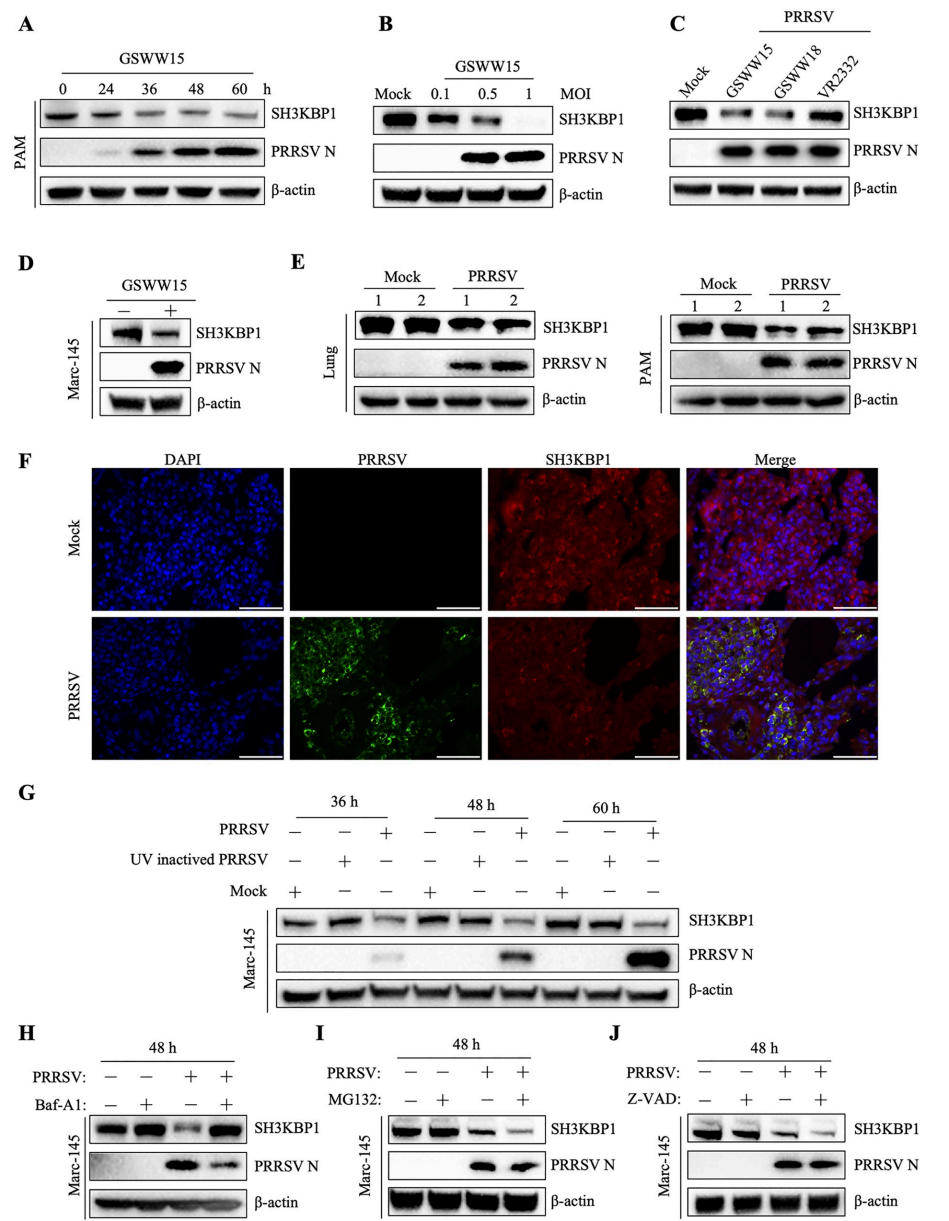
PRRSV infection down-regulates SH3KBP1 expression in PAM and Marc-145 cells. **(A)** PAM cells were infected with PRRSV (MOI = 0.1) for 24, 36, 48 and 60 h. The cell lysates were collected and the expression of SH3KBP1 was detected by Western blot. **(B)** PAM cells were infected with PRRSV (MOI = 0.1, 0.5, 1). Samples were collected 36 h after infection and the protein level of SH3KBP1 was detected. **(C)** PAM cells were infected with the highly pathogenic representative strain (GSWW15), NADC30-like strain (GSWW18), and vaccine strain (VR2332) (MOI = 0.1). Samples were collected 36 h after infection and the protein level of SH3KBP1 were detected. **(D)** Marc-145 cells were mock treated or infected with GSWW15 (MOI = 0.1), cell lysates were collected at 36 h to detect SH3KBP1 protein level by Western blot. **(E)** The lung tissues and PAM cells of the PRRSV-infected group and uninfected group were randomly collected to detect the expression level of SH3KBP1 protein. Four 5-week-old specific-pathogen-free (SPF) pigs were randomly assigned to two experimental groups: the infected group (n  =  2) and the uninfected group (n  =  2). Pigs were intranasally injected with PRRSV GSWW15 strain (2 ml of TCID_50_ = 10 ^5.5^/100 μL virus stock per pig) and DMEM as a control in the two experimental groups, respectively. When all the infected pigs displayed the clinical signs after the challenge, all the experimental pigs were euthanized, the lungs were collected, PAM cells were isolated, and the protein level of SH3KBP1 was detected by Western blot. **(F)** *In vivo* immunofluorescence staining was used to detect endogenous SH3KBP1 and PRRSV-infected lung tissue of experimental pigs. Sections were prepared from the formalin-fixed, paraffin-embedded lung tissues and incubated with primary antibodies against SH3KBP1 and PRRSV N protein, followed by probing with the appropriate secondary antibodies. The fluorescence was visualized after nuclei were stained with DAPI. Scale bars, 50 μm. **(G)** Marc-145 cells were inoculated with PRRSV or UV-inactivated PRRSV, respectively. Samples were collected 36, 48, and 60 h after infection, and the SH3KBP1 protein levels were detected by Western blot. **(H-J)** Marc-145 cells were treated with Baf-A1 (100 nM) (H), MG132 (5 μM) (I), Z-VAD (10 μM) (J) and DMSO, inoculated with PRRSV (MOI = 0.1), and samples were collected at 48 h. Cell lysates were then subjected to Western blot with the indicated antibody. Data are representative of three independent experiments.

Given that PAM is the cell targeted by PRRSV in the natural host, we investigated the effect of PRRSV infection on SH3KBP1 expression in PAMs. Western blot results showed that endogenous SH3KBP1 was significantly down-regulated in PAMs upon PRRSV infection in a time and dose-dependent manner (Fig 7A and 7B). To investigate whether the downregulation of SH3KBP1 by PRRSV is strain specific, we inoculated PAMs with GSWW15 (HP-PRRSV-like strain), GSWW18 (NADC30-like strain) and vaccine strain VR2332. Infection with all three PRRSV strains results in reduced levels of endogenous SH3KBP1 protein (Fig 7C). The degradation of SH3KBP1 by PRRSV infection was also observed in Marc-145 cells (Fig 7D). We also investigated whether PRRSV infection affected the mRNA level of SH3KBP1. The results showed that PRRSV infection did not affect SH3KBP1 mRNA levels (S5A Fig).

To test whether PRRSV infection induces SH3KBP1 reduction *in vivo*, we performed animal studies. Western blot results showed that the expression level of SH3KBP1 in the lungs and PAMs of PRRSV-infected piglets was significantly lower than mock-infected pigs (Fig 7E). Additionally, immunofluorescence analysis demonstrated an overall decrease in SH3KBP1 expression (highlighted in red) in pig lungs upon PRRSV infection compared to mock-infected piglets (Fig 7F). To verify whether the downregulation of SH3KBP1 was dependents on viral replication, Marc-145 cells were treated with UV-inactivated PRRSV. Notably, UV-inactivated PRRSV did not reduce SH3KBP1 protein levels (Fig 7G), suggesting that downregulation of SH3KBP1 during PRRSV infection was caused by invading virions or intermediates produced during viral replication. In summary, these findings demonstrated that PRRSV infection induces degradation of SH3KBP1 both *in vitro* and *in vivo*.

The ubiquitin-proteasome and autophagy-lysosome pathways are the primary systems that regulate protein degradation in eukaryotic cells. To determine the degradation pathway of SH3KBP1, we treated Marc-145 cells with proteasome inhibitor MG132, lysosome inhibitor Bafilomycin A1 (Baf-A1) and apoptotic inhibitor Z-VAD. Baf-A1 treatment partially restored SH3KBP1 protein levels in PRRSV-infected cells, while Z-VAD and MG132 did not (Fig 7H–7J), indicating that PRRSV induces SH3KBP1 degradation through the autophagic pathway. Moreover, we found that SH3KBP1 was also degraded upon SeV infection through the autophagic pathway (S5G–S5J Fig).

### PRRSV NSP2 (^453^PVPAPR^458^) amino acid sequence is the key region for degradation of SH3KBP1

**Fig 8:**
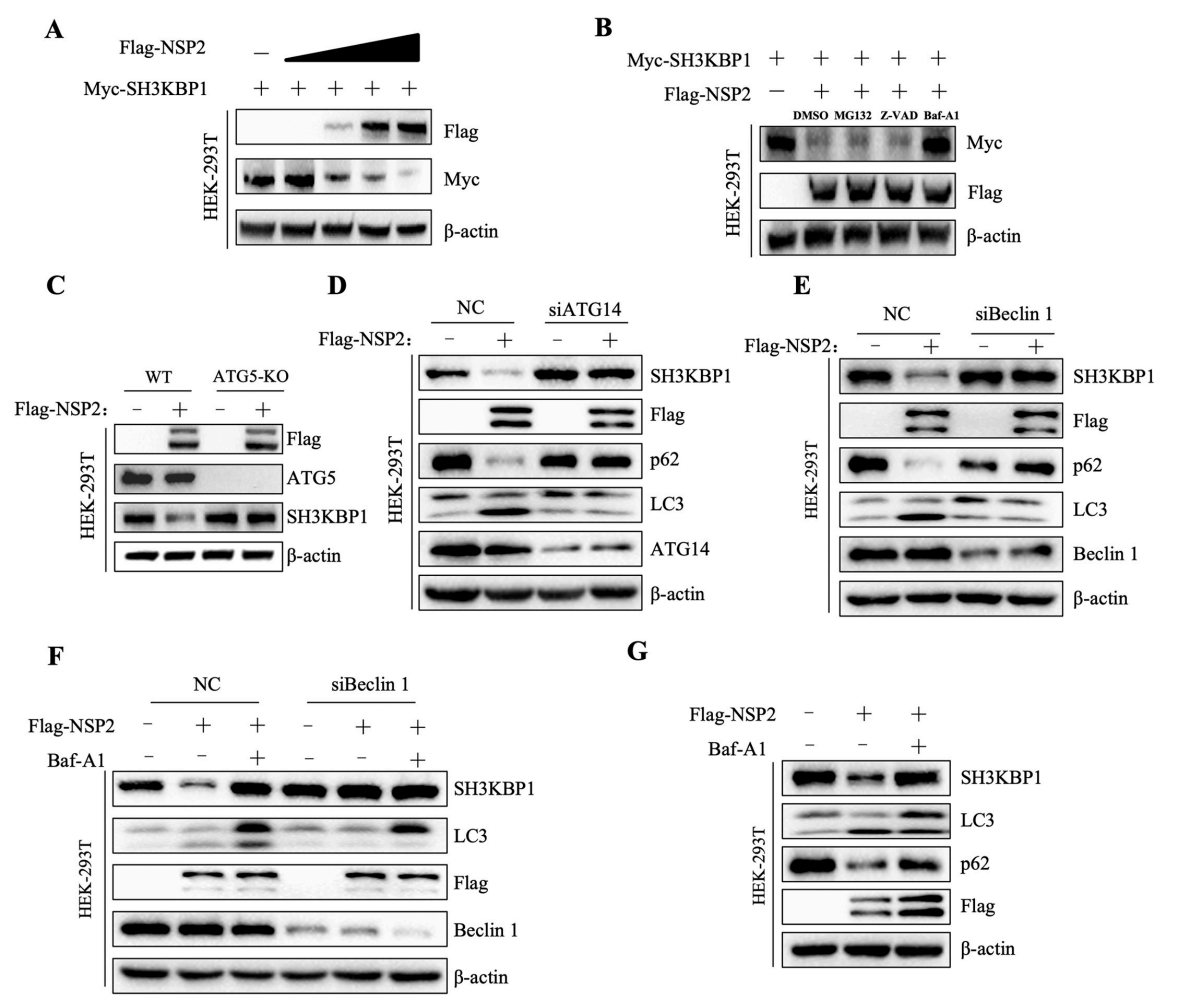
PRRSV NSP2 protein induces SH3KBP1 degradation via the autophagy pathway. **(A)** HEK-293T were transfected with Myc-SH3KBP1, together with Flag-EV or different concentrations of Flag-NSP2 for 24 h. The cells were harvested for Western blot. **(B)** HEK-293T cells were transfected with Flag-NSP2 and Myc-SH3KBP1 and then treated with Baf-A1, MG132, Z-VAD and DMSO, respectively. At 12 h, the cells were harvested for Western blot to detect SH3KBP1 protein levels. **(C)** WT and ATG5-KO cells were transfected with Flag-NSP2, and then collected at 24 h for Western blot. **(D-E)** HEK-293T cells were transfected with control (NC) or siRNA and Flag-NSP2 for 30 h. The cell lysates were subjected to Western blot with the indicated antibodies. **(F)** After interfering with HEK-293T cells with siNC/siBeclin1, Flag-EV or Flag-NSP2 was transfected for 12 h. Before harvest, the cells were treated with DMSO or Baf-A1 for 12 h. The cell lysates were subjected to Western blot with specific antibodies. **(G)** HEK-293T cells were transfected with Flag-EV or Flag-NSP2 for 12 h. Before harvesting, the cells were treated with DMSO or Baf-A1 for 12 h. The cell lysates were used for Western blot with the indicated antibodies. Data are representative of three independent experiments.

We screened non-structural proteins of PRRSV and found that NSP2 and NSP12 promoted SH3KBP1 degradation (S6A Fig). Subsequently, we performed Co-IP assays and found that only NSP2 interacted with SH3KBP1, so we focused on NSP2 in our study (S6B Fig). Western blot analysis results showed that increasing NSP2 expression led to a gradual decrease in SH3KBP1 protein levels, indicating that NSP2 promoted SH3KBP1 degradation in a dose-dependent manner (Fig 8A). Additionally, qPCR results showed that overexpression of NSP2 did not affect mRNA level of SH3KBP1 (S5B Fig). Our results demonstrated that NSP2-mediated degradation of SH3KBP1 could be largely reversed by treatment with the autophagy inhibitors Baf-A1, but not by the proteasome inhibitor MG132 or the caspase inhibitor Z-VAD (Fig 8B). To further verify this result, we transfected Flag-tagged NSP2 into ATG5-KO cells and observed that NSP2 did not decrease SH3KBP1 expression in these cells (Fig 8C). Additionally, we found that in cells where autophagy genes (ATG14 and Beclin 1) were knocked down by siRNAs, NSP2 did not induce the degradation of SH3KBP1 (Fig 8D and 8E). Moreover, we treated Beclin 1 knockdown cells with Baf-A1 and found that the SH3KBP1 protein level was not further restored (Fig 8F). In ATG5 KO cells, NSP2 overexpression did not altered the protein levels of RIG-I signaling molecules (S6C Fig). It has been previously reported that NSP2 induces autophagy [32–35]. Our results confirmed that transfection of NSP2 increased LC3II levels and decreased p62 protein levels (Fig 8G). We examined the mRNA levels of PRRSV after treatment with Baf-A1. The results demonstrated that Baf-A1 inhibited virus replication without affecting virus adsorption and internalization (S5D–S5F Fig). These findings indicated that the restored expression of SH3KBP1 by Baf-A1 was not due to its inhibition of virus entry.

**Fig 9:**
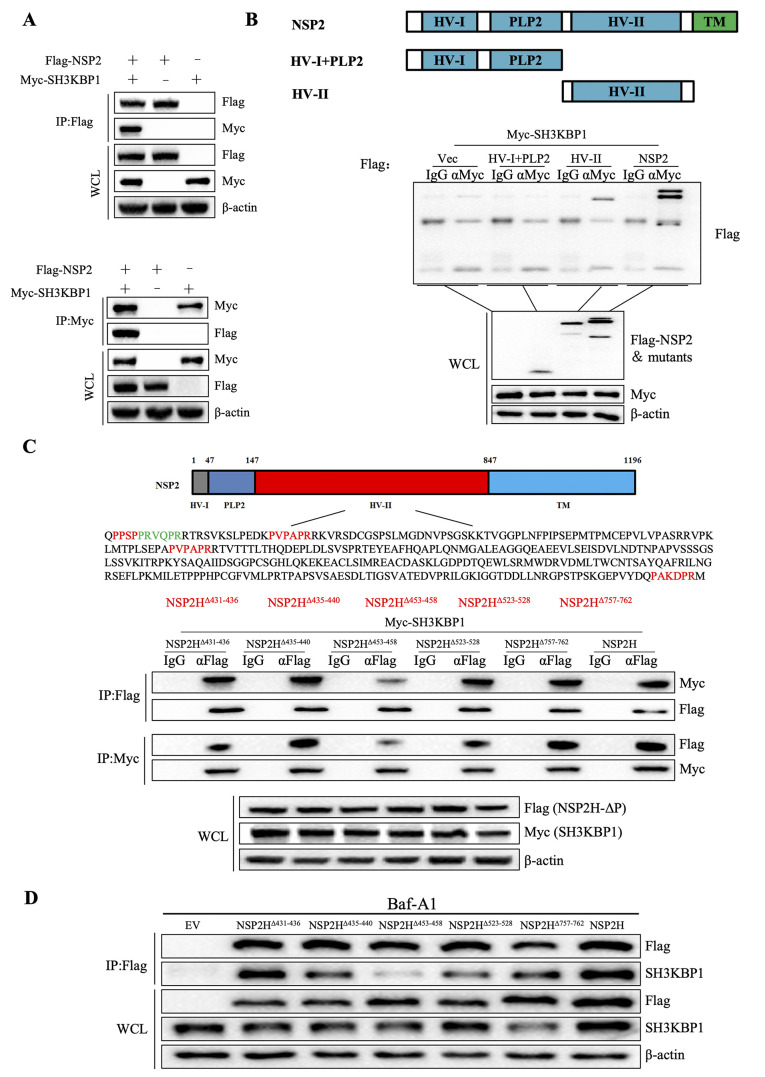
The amino acid sequence ^453^PVPAPR^458^ in PRRSV NSP2 is the key region for its interaction with SH3KBP1. **(A)** HEK-293T cells were transfected with Flag-NSP2 and Myc-SH3KBP1, and WCL collected at 24 h were subjected to IP with anti-Flag antibodies or anti-Myc antibodies. **(B)** Schematic of full-length NSP2 and its truncation. HEK-293T cells were transfected with Flag-NSP2 full length or truncations and Myc-SH3KBP1 for 24 h, followed by Co-IP and Western blot analysis with indicated antibodies. **(C)** Schematic of full-length NSP2 and PXXXPR sequences. HEK-293T cells were transfected with Flag-NSP2-2H^Δ^ and Myc-SH3KBP1, and WCL collected at 24 h were subjected to IP with anti-Flag antibodies or anti-Myc antibodies. **(D)** HEK-293T cells were transfected with Flag-NSP2-2H^Δ^, before harvesting, the cells were treated with DMOS or Baf-A1 for 12 h. The cell lysates were used for Co-IP and Western blot analysis with the indicated antibodies. Data are representative of three independent experiments.

The above results confirmed that NSP2 promoted the autophagic degradation of SH3KBP1. To validate the interaction between SH3KBP1 and NSP2, we co-expressed Flag-tagged NSP2 with Myc-tagged SH3KBP1 in HEK-293T cells and performed co-immunoprecipitation (Co-IP) assays using anti-Flag and anti-Myc antibodies. As shown in Fig 9A, co-immunoprecipitation confirmed that Flag-tagged NSP2 co-precipitates with Myc-tagged SH3KBP1. We constructed NSP2 truncations and performed Co-IP assays. The results demonstrated that NSP2 interacts with SH3KBP1 via its HV-II domain (Fig 9B).

Given that the SH3 domain of SH3KBP1 binds polyproline-arginine motifs, and NSP2 contains five such motifs (PXXXPR), we sought to identify which motifs are essential for this interaction. We constructed a series of Flag-tagged NSP2 expression plasmids with deletions of different PXXXPR motifs. These mutants and Myc-tagged SH3KBP1 were co-transfected into HEK-293T cells, following by Co-IP assays with anti-Flag and anti-Myc antibodies. The results demonstrated that NSP2 could still interact with SH3KBP1 when only one PXXXPR region was deleted (Fig 9C). We also analyzed the interaction between NSP2 mutants and endogenous SH3KBP1. The Co-IP results showed that deletion of the 453–458 amino acid sequence diminished their binding (Fig 9D). Subsequently, we analyzed the colocalization coefficient index of wild type and mutant NSP2 with SH3KBP1. The results showed that deletion of amino acids 453–458 disrupted the colocalization of NSP2 and SH3KBP1. Similarly, deletion of amino acids 523–528 also decreased their colocalization, while other deletions had no significant effect on their interaction (S6D Fig). These results indicated that the absence of a single PXXXPR region dose not abolish their interaction. However, deletion of the PAPVPR sequence at residues 453–458 significantly reduced the interaction, suggesting this motif is crucial for the NSP2-SH3KBP1 interaction.

**Fig 10:**
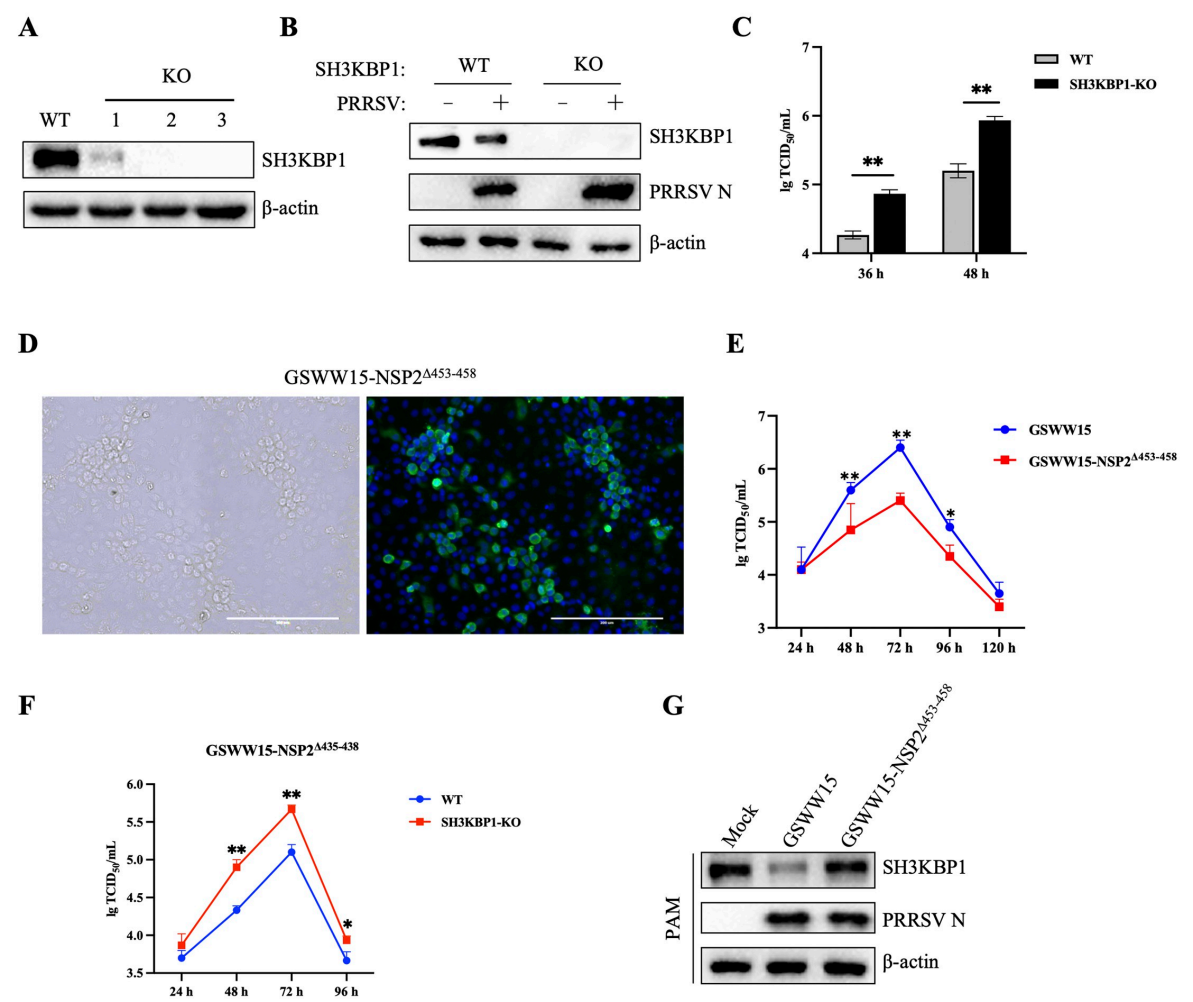
Construction of GSWW15-NSP2^Δ453–458^. **(A)** Deficiency of SH3KBP1 in the Marc-145 cells was confirmed by Western blot analysis with anti-SH3KBP1 antibody. **(B)** WT and SH3KBP1-KO Marc-145 cells were infected with GSWW15 (MOI = 0.1), samples were collected 36 h after infection, and the expression of N protein was detected by Western blot. **(C)** Determination of GSWW15 virus titers in WT and SH3KBP1-KO Marc-145 cell. WT and SH3KBP1-KO Marc-145 cells were infected with GSWW15 (MOI = 0.1), and virus titers were measured at the indicated times post-infection. **(D)** Immunofluorescence analysis of rescued GSWW15-NSP2^Δ453–458^ mutant that was constructed basis on GSWW15 strain. Marc-145 cells were infected with the rescue mutant strain (MOI = 0.1) for 36 h. PRRSV protein N was detected using SR30 and an FITC-488-conjugated secondary antibody. Scale bars, 200 μm. **(E)** Multistep virus growth curves of GSWW15 and GSWW15-NSP2^Δ453–458^ (MOI = 0.1). Marc-145 cell were infected with GSWW15 and GSWW15-NSP2^Δ453–458^, and virus titers were measured at the indicated times post-infection. **(F)** Multistep virus growth curves of GSWW15-NSP2^Δ453–458^ (MOI = 0.1) in WT and SH3KBP1-KO Marc-145 cells. WT and SH3KBP1-KO Marc-145 cell were infected with GSWW15-NSP2^Δ453–458^, and virus titers were measured at the indicated times post-infection. **(G)** Marc-145 cells were mock infected or infected with GSWW15 and GSWW15-NSP2^Δ453–458^ (MOI = 0.1), cell lysates were collected at 48 h to detect SH3KBP1 protein level by Western blot. Data are representative of three independent experiments.

We constructed SH3KBP1 knockout Marc-145 cells and assessed PRRSV replication in both WT and knockout (KO) cells (Fig 10A). The results showed that the N protein level and virus titer were higher in SH3KBP1 KO cells compared to WT cells (Fig 10B and 10C). To further explore the role of the NSP2 453–458 amino acid sequence, we constructed a PRRSV GSWW15-NSP2^Δ453–458^ mutant strain using reverse genetic (Fig 10D). We assessed the in vitro growth characteristics of this mutant and the wild-type GSWW15 strain in Marc-145 cells. The GSWW15-NSP2^Δ453–458^ mutant displayed significantly delayed growth kinetics compared to the wild-type strain (Fig 10E). Compared with the WT cells, the replication ability of GSWW15-NSP2Δ453–458 was enhanced in the SH3KBP1-KO cells (Fig 10F). Additionally, the mutant strain exhibited a reduced ability to degrade SH3KBP1 (Fig 10G). These findings indicate that the NSP2 ^453^PVPAPR^458^ amino acid sequence is critical for viral attenuation and SH3KBP1 degradation.

## Discussion

**Fig 11:**
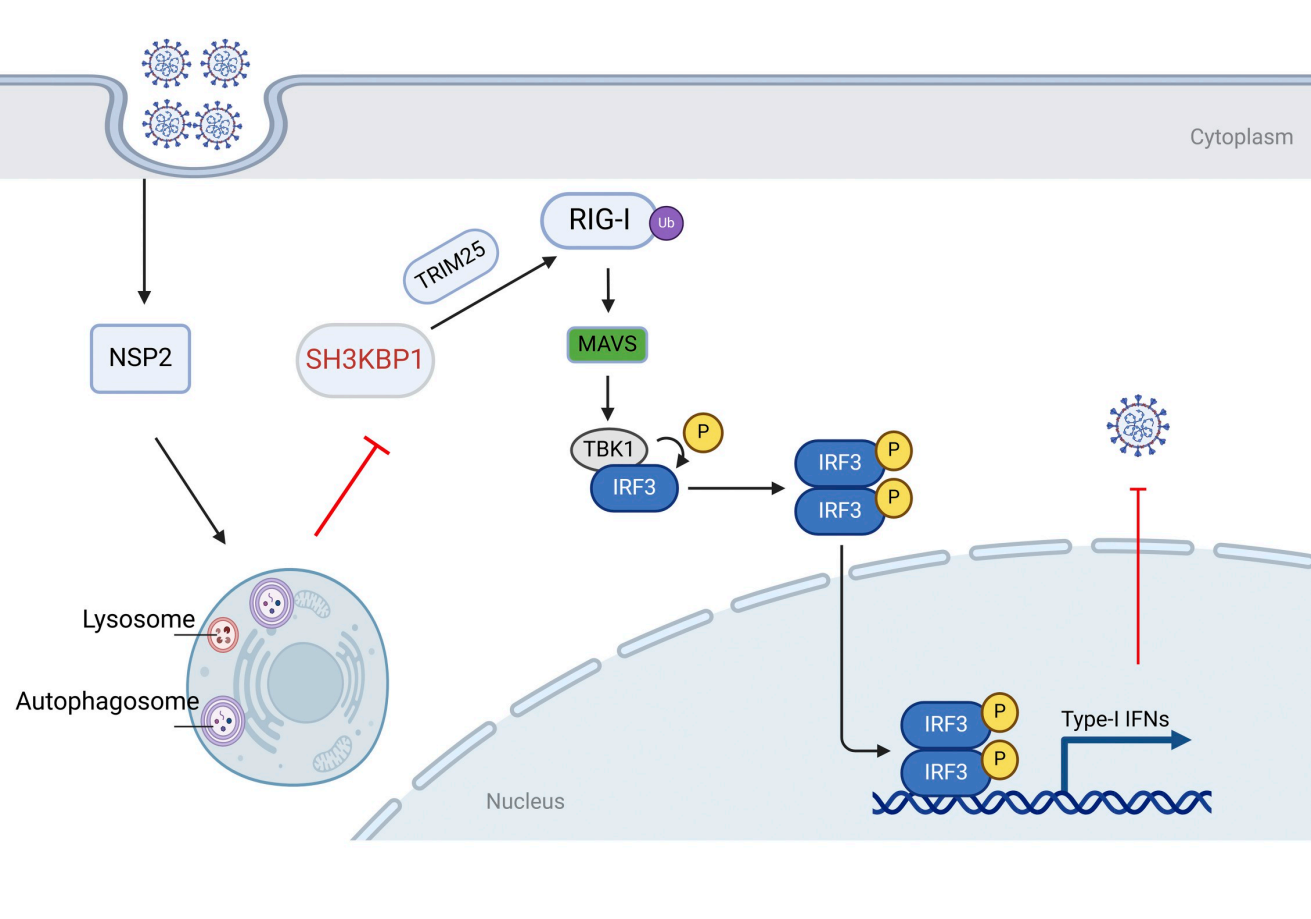
Schematic model of the mechanism by which SH3KBP1 impairs PRRSV replication, created in BioRender. In the resting state, SH3KBP1 enhances the K63-linked polyubiquitination of RIG-I by recruiting TRIM25, thereby promoting IFN-β production. After viral infection, PRRSV NSP2 binds to and degrades SH3KBP1 via the autophagy pathway, inhibiting the IFN-β signaling pathway and thus facilitating viral replication.

PRRS is a serious threat to the global swine industry. However, its pathogenic mechanism is not fully understood. Rapid genetic variations within the NSP2 region indicated that it plays an essential role in PRRSV biology and pathogenesis. In this study, we reveal a novel role for SH3KBP1 in regulating the IFN-I signaling pathway. SH3KBP1 promoted the protein level of RIG-I and inhibits PRRSV replication *in vitro*. It recruited TRIM25 and enhanced K63-linked polyubiquitination of RIG-I to promote signal transduction. At the same time, PRRSV NSP2 degraded SH3KBP1 through autophagic pathway and antagonized the antiviral effect of SH3KBP1 (Fig 11). This study illustrated a regulatory role of SH3KBP1 in the innate immunity and provided a novel mechanism by which PRRSV antagonizes the host innate immune response.

The innate immune system is the first line to protect the host against viral invasion. As an adaptor protein, SH3KBP1 selectively controls the spatial and temporal assembly of multi-protein complexes that transmit intracellular signals and are involved in regulating many cellular processes. Previously, it has been reported to regulate replication of some viruses, however its role in innate immunity is not fully understood [27–29]. In this study, we found that overexpression of SH3KBP1 up-regulated the expression of IFN-β and ISGs, whereas its deficiency restricted it (Fig 2). In addition, *Sh3kbp1*^-/-^ mice challenged with VSV produced a lower level of IFN-β and pro-inflammatory cytokines *in vivo* compared to *Sh3kbp1*^+/+^ mice and exhibited lower survival rate (Fig 3B–3C). Our results suggest a broad antiviral role for SH3KBP1, which restrains viral replication by upregulating the IFN expression.

RLR signaling is tightly regulated to ensure proper activation in response to viral infections. K63-linked ubiquitination of RIG-I is essential for its activation. Histone demethylase LSD1 promotes K63-linked polyubiquitination of RIG-I via TRIM25 [36]. The RNA-binding protein ZFP36 promotes K63-linked polyubiquitination of RIG-I, thereby facilitating RIG-I activation during viral infection [37]. In this study, we found that overexpression of SH3KBP1 led to increased levels of RIG-I at protein level (Figs 4A, 4D and S3A). In vivo ubiquitination assay showed that overexpression of SH3KBP1 significantly increased K63-linked polyubiquitination of RIG-I (Fig 5). Co-IP results showed that overexpression of SH3KBP1 enhanced the binding between TRIM25 and RIG-I (Fig 6C), whereas SH3KBP1 deficiency reduced their interaction. These results revealed the underlying mechanism by which SH3KBP1 enhances IFN signaling.

During the evolution of viruses, there are a variety of strategies to evade innate immune responses, such as the degradation of antiviral proteins through their own proteins. PRRSV E protein degrades DDX10 by promoting SQSTM1-mediated selective autophagy and escapes host antiviral innate immunity [38]. The NSP3 of PRRSV was demonstrated to induce the proteasome-dependent degradation of porcine intrinsic virus-restriction factor IFITM1 upon the virus infection. [39]. PRRSV NSP1-mediated the CREB-binding protein (CBP) degradation inhibits the recruitment of CBP for enhanceosome assembly, leading to the block of IFN response [40]. PRRSV NSP11 degrades ISG15 relied on autophagy-lysosome system to promote PRRSV replication [41]. The PRRSV NSP2 can prevent the degradation of the IκB kinase complex α (IκB kinase α, IKKα) by polyubiquitination, thereby impeding the production of type I IFN [42]. In this study, we found that PRRSV NSP2 can interact with SH3KBP1 and degrade SH3KBP1 through the autophagic pathway (Fig 8 and 9), thereby inhibiting the antiviral response mediated by SH3KBP1 and ultimately conducive to the replication of the virus.

Autophagy can promote or inhibit virus replication, depending on the virus type, cell type, or cellular environment [43–47]. PRRSV infection induces autophagy (S5C Fig), which in turn promotes its replication [35]. In this study, we found that PRRSV infection-induced SH3KBP1 degradation was restored by treatment with the autophagy inhibitor Baf-A1 but not the protease inhibitor MG132 or the caspase inhibitor Z-VAD (Fig 7H–7J). By screening the NSPs, we found that NSP2 and NSP12 promoted the degradation of SH3KBP1 (S6A Fig). Since only NSP2 interacted with SH3KBP1, we focused on NSP2 in this study. We demonstrated that NSP2 promoted autophagic degradation of SH3KBP1. NSP2 could not promote SH3KBP1 degradation in autophagic component deficient cells (Fig 8C–8E). PRRSV NSP2 has been previously reported to induce autophagy [32]. Mechanistically, NSP2 induces ER-stress, by interacting with the 78 kDa glucose-regulated protein 78 (GRP78). In addition, PRRSV NSP2 interacts with and degrades Golgi reassembly and stacking protein 65 (GRASP65) through its papain-like cysteine protease 2 activity, leading to Golgi apparatus fragmentation [34]. This process promotes autophagy, facilitating viral replication. In this study, we revealed that PRRSV utilized autophagic process to degrade SH3KBP1, thereby counteracting the host’s antiviral IFN signaling.

Interestingly, we found that the absence of the NSP2 453–458 amino acid sequence severely affected the interaction between the NSP2 and SH3KBP1 (Fig 9C–9D). To verify the function of NSP2 453–458 amino acid sequence, we successfully constructed a PRRSV GSWW15-NSP2^Δ453–458^ mutant strain by reverse genetic technique. Compared to GSWW15, GSWW15-NSP2^Δ453–458^ mutant strain has an attenuated growth kinetic (Fig 10E). The immunological properties of the mutant strain to the host need to be further studied. At the same time, we found that the ability of GSWW15-NSP2^Δ453–458^ mutant strain to degrade SH3KBP1 was reduced to some extent (Fig 10G). In summary, we show that the NSP2 ^453^PVPAPR^458^ amino acid sequence is a key region for the interaction between NSP2 and SH3KBP1, which is a key region for the degradation of SH3KBP1 by NSP2.

To sum up, our study demonstrates SH3KBP1 promotes K63-linked polyubiquitylation of RIG-I by recruiting TRIM25, thereby positively regulating IFN-I signaling pathway and inhibiting PRRSV replication. In addition, PRRSV and NSP2 degrade SH3KBP1 through autophagy pathway, thus antagonizing the host antiviral response. GSWW15-NSP2^Δ453–458^ mutant strain showed an attenuated virulence and a decreased degradation of SH3KBP1. Our findings provide insight into the potential role of SH3KBP1 in innate immunity and contribute to a better understanding of PRRSV infection and its pathogenesis.

## Materials and methods

### Ethics statement

All animals were strictly handled in accordance with the Good Animal Practice of the People’s Republic of China Animal Ethics Procedures and Guidelines. All mouse studies have been approved by the Animal Ethics Committee of Lanzhou Institute of Veterinary Medicine, Chinese Academy of Agricultural Sciences (Permit No. LVRIAEC-2023-089).

### Cells and viruses

Human embryonic kidney 293T cells (HEK-293T), human embryonic kidney 293 cells (HEK-293), Marc-145 cell, SH3KBP1 KO cells and WT cells, ATG5 KO cells and WT cells were maintained in our laboratory, while TRIM25 KO cells and WT cells were constructed by Guangzhou Yuanjing Biotechnology Co., Ltd. Cells were cultured in Dulbecco’s modified Eagle’s medium (DMEM; Gibco), supplemented with 10% fetal bovine serum (FBS), 100 U/mL penicillin, and 100 mg/mL streptomycin at 37°C with 5% CO_2_. PAMs were prepared from lung lavage samples from 4-week-old healthy piglets, grown in RPMI 1640 medium containing 10% fetal bovine serum, and maintained at 37°C with 5% CO_2_. All cells were negative for mycoplasma. Sendai virus (SeV), and PRRSV GSWW15 (GenBank accession no. KX767091.1), GSWW18 (GenBank accession no. OP764591.1), VR2332 were stored in our laboratory.

### Mice

*Sh3kbp1*^-/-^ mice on the C57BL/6 background were purchased from Cyagen. Mice genotype identification was performed by polymerase chain reaction (PCR) analysis of DNA isolated from the tail using the following primers: Sh3kbp1-F1, 5’-ATCTGCTCTATGCAACCAATCTCT-3’; Sh3kbp1-R1, 5’-CTTTGATGCATGATTCTCTGGGTC-3’.

### Antibodies and reagents

Anti-HA (3724), anti-Myc (2276), anti-ubiquitin (3936), anti-p-TBK1 (5483), anti-TBK1 (3504), anti-p-IRF3 (4947), anti-K48-linkage Specific Polyubiquitin (8081), anti-K63-linkage Specific Polyubiquitin (5621), anti-IRF3 (11904), anti-ATG5 (2630), anti-TRIM25 (13773), anti-RIG-I (3743), anti-SH3KBP1 (12304), anti-mouse IgG-HRP-linked Antibody (7076), anti-rabbit IgG-HRP-linked Antibody (7074) were obtained from Cell Signaling Technology. Anti-MAVS (sc-166583) was purchased from Santa Cruz Biotechnology. Anti-Interferon beta (CY6690), anti-RANTES (CY7179), anti-HSP90(CY5548), anti-Lamin B1 (AB0054) were obtained from Abways technology. Anti-ISG15 (A2416), anti-ATG14 (A7526), anti-Beclin 1 (A21191) were obtained from ABclonal. Goat Anti-Mouse IgG H&L (FITC) (ab6785), and Goat Anti-Rabbit IgG H&L (Cy3) preadsorbed (ab6939) were obtained from Abcam. Anti-β-Actin (A5441), anti-Flag (F1804), and anti-pig IgG-HRP-linked Antibody (SAB3700434) were obtained from Sigma Aldrich. SR30 monoclonal antibody against N protein was purchased from RTI LLC (Brookings, SD, USA). MG132 (HY-13259), Z-VAD-FMK (HY-16658B), Hygromycin B(HY-B0490) and Bafilomycin A1 (Baf-A1; HY-100558) were purchased from MedChemExpress. Additionally, mouse IgG (A7028), and rabbit IgG (A7016) and Cell Counting Kit-8(C0038), are available from Beyotime. Human Regulatory Activation Protein (Rantes) ELISA kit (ml058605) and mouse interferon beta ELISA kit (CK-E10284) were obtained from Shanghai Enzyme-linked Biotechnology. Puromycin (A1113803) and NE-PER Nuclear and Cytoplasmic Extraction Reagents (78833) are purchased from Thermo Fisher.

### Plasmids

Myc-tagged SH3KBP1 plasmids were constructed by inserting full-length SH3KBP1 cDNAs synthesized from total RNAs of Marc-145 or HEK-293T into pCMV-Myc vectors. Full-length PRRSV NSP2 was amplified from the PRRSV genome and cloned into the pCDNA3.1 vector to generate Flag-tagged protein expression plasmids using standard molecular biology techniques.

### RT-qPCR

Total cellular RNA was extracted using an RNA extraction kit (OMEGA). cDNA was synthesized using the RT master mix reagent kit (TaKaRa), or probe qPCR Premix Ex Taq (TaKaRa) was used for quantitative real-time PCR. SYBR green real-time PCR was used to analyze gene expression. Data were normalized to GAPDH expression level. All RT-qPCR primers are presented in Table 2.

**Table 2:** Primers for RT-qPCR.

Primer	Sequence
GAPDH-F(HEK-293T)	GTCGTCGACAACGGCTCCG
GAPDH-R(HEK-293T)	ATTGTAGAAGGTGTGGTGC
IFN-β-F(HEK-293T)	CACGACAGCTCTTTCCATGA
IFN-β-R(HEK-293T)	AGCCAGTGCTCGATGAATCT
ISG15-F(HEK-293T)	ATGGGCTGGGACCTGACGG
ISG15-R(HEK-293T)	TTAGCTCCGCCCGCCAGGCT
ISG54-F(HEK-293T)	CACCTCTGGACTGGCAATAGC
ISG54-R(HEK-293T)	GTCAGGATTCAGCCGAATGG
ISG56-F(HEK-293T)	TCATCAGGTCAAGGATAGTC
ISG56-R(HEK-293T)	CCACACTGTATTTGGTGTCTAGG
RANTES-F(HEK-293T)	ATGAAGGTCTCCGCGGCACG
RANTES-R(HEK-293T)	CTAGCTCATCTCCAAAGA
OAS-F(HEK-293T)	GGAGCCAGCATCGTACCCC
OAS-R(HEK-293T)	ATCCCTTAGGTCTGTGCCCC
GAPDH-F(Marc-145)	CAAGAAGGTGGTGAAGCA
GAPDH-R(Marc-145)	AAGGTGGAAGAGTGGGTG
IFN-β-F(Marc-145)	TGCTCCAGAACATCTTCGCT
IFN-β-R(Marc-145)	GTGACTGTACTCCTTGGCCT
ISG15-F(Marc-145)	CTGAAGGCAAAGATCGCCCA
ISG15-R(Marc-145)	GTCGTTCCTCACCAGGATGC
ISG56-F(Marc-145)	TGGACAGGAAGCTGAAGGAG
ISG56-R(Marc-145)	GCCCTTTTGTAGCCTCCTTG
RANTES-F(Marc-145)	GCCTGTTTCTGCTTGCTCTT
RANTES-R(Marc-145)	TTTCATCATGTTGGCCAGGC
IL-6-F(Mouse)	TCTGCAAGAGACTTCCATCCAGTTGC
IL-6-R(Mouse)	AGCCTCCGACTTGTGAAGTGGT
GAPDH-F(Mouse)	ACGGCCGCATCTTCTTGTGCA
GAPDH-R(Mouse)	ACGGCCAAATCCGTTCACACC
IL-1β-F(Mouse)	TGGCAACTGTTCCTG
IL-1β-R(Mouse)	GGAAGCAGCCCTTCATCTTT
TNF-α- F(Mouse)	CATCTTCTCAAAATTCGAGTGACAA
TNF-α- R(Mouse)	TGGGAGTAGACAAGGTACAACCC

### Western blot

Target proteins were separated by SDS-PAGE and transferred onto polyvinylidene fluoride (PVDF) membranes. Subsequently, membranes were blocked with 5% skim milk at room temperature for 2 h, incubated with appropriate primary antibody at 4°C overnight, and incubated with secondary antibody at room temperature for 1 h. Blots were treated with chemiluminescent ECL substrate (34080) and imaged using digital capture (Chemdoc MP, America).

### Co-IP

The plasmid was transfected into HEK-293T cells or Marc-145 cells. 24 h after transfection, cells were lysed with IP lysis buffer containing protease inhibitors and phenylmethylsulfonyl fluoride (PMSF) at 4°C for 2–4 h. The lysate was centrifuged, the supernatant was collected, and incubated with a suitable antibody or control IgG at 4°C overnight. Protein A/G magnetic beads were added to the antibody complex and rotated at 4°C for 2 h. After washing the magnetic beads with PBS containing Tween 20 (PBST) for five times, the beads were added to the protein loading buffer, thoroughly mixed, and boiled, and the samples were analyzed by Western blot.

### Indirect immunofluorescence assays and confocal

Marc-145 cells and HEK-293T cells were transfected with indicated plasmids for 24 h and then fixed with 4% paraformaldehyde for 30 min. Subsequently, cells were washed with PBS for three times, followed by permeabilization with a 0.1% solution of Triton X-100. Fixed and permeabilized monolayers were incubated with 5% BSA at room temperature for 2 h and incubated with the corresponding primary antibodies at 4°C overnight. Cells were washed with PBS for three times. Then, cells were incubated with fluorescent labeled secondary antibodies at room temperature for 1 h. Finally, nuclei were counterstained with 4’, 6-diamino-2-phenylindole (DAPI) for 15 min. Fluorescence images were acquired using laser scanning confocal immunofluorescence microscopy (TCS SP8, Germany).

### ELISA

The concentrations of IFN-β or RANTES in culture supernatant and serum were detected by ELISA kit according to the manufacturer’s instructions. Briefly, add 50 μL of the sample to be tested, mix it gently, then add 100 μL of enzymic reagent, incubate at 37°C for 60 min, and wash it with TBST for 5 times. Add 50 μL color developer A to each well, then add 50 μL color developer B, gently shake and mix, hide from light for 15 min at 37°C. The termination process was terminated by adding a termination solution of 50 μL per well, and the optical density of 450 nm (OD450) was measured on a microplate reader (BioRad).

### RNAi

Chemically synthesized siRNAs used in RNA interference (RNAi) assays were manufactured by JTS (Wuhan China). The knockdown of endogenous SH3KBP1 was carried out by transfection of the indicated SH3KBP1 siRNA into cells using Lipofectamine 3000 (Invitrogen). The siRNA sequences targeted for siSH3KBP1 are as follow siSH3KBP1 sense, 5’-GGCACAGAAUGAUGAUGAATT-3’ and siSH3KBP1 antisense, 5’-UUCAUCAUCAUUCUGUGCCTT-3’.

### Luciferase reporter assay

HEK-293T cells were seeded on a 24-well plate. When the cell density reached 70–80%, the double luciferase reporter plasmid, pRL-TK, and Myc-EV or Myc-SH3KBP1 plasmid were co-transfected into HEK-293T cells using Lipofectamine 2000 (Invitrogen). After treatment as indicated, cells were harvested and the reporter gene activity was detected using a dual-specific luciferase assay kit (Promega, Madison, WI).

### Rescue of PRRSV mutants by reverse genetics

The GSWW15 strain isolated in our laboratory was sequenced and synthesized in sections. According to the structural and non-structural proteins encoded by the PRRSV genome, the genome is divided into A and B by *Nhe* I. The two fragments were constructed into the pBR323 vector preserved in the laboratory to form two plasmids, pGS-A and pGS-B. Deletion of specific regions of pGS-A plasmid was performed using the Vazyme point mutation kit. pGS-A and pGS-B were digested by enzymes, and the fragments was recovered to form the plasmid pGS-AB. All mutant constructs were validated by sequencing. Subsequently, *Acl* I-linearized mutant plasmids were transfected into BSR/T7 cells, following the manufacturer’s instructions, using Lipofectamine 2000. At 72 h post-transfection, the cells were harvested and passaged on Marc-145 cells. After 3 rounds of passaging, the copy number of mutant virus was determined by qPCR.

### Cell viability assay

Cells viability was determined using CCK-8 assays. Briefly, cells were inoculated into a 96-well plate and overexpressed or interfered with SH3KBP1 for 60 h. 10 μL CCK-8 solution was added to each well. After 2 h of incubation, the optical density of each well was measured at a wavelength of 450 nm.

### CRISPR/Cas9 knockout

Double-stranded oligonucleotides corresponding to the target sequences were cloned into the lenti-CRISPR-V2 vector and co-transfected packaging plasmids into HEK-293 cells. Two days after transfection, the viruses were harvested, ultra-filtrated. Puromycin or Hygromycin B was used for KO cell selection, and immunoblot analysis was performed to determine the knockdown efficiency. The corresponding gRNA oligonucleotide sequences were as follows: human SH3KBP1 gRNA:(1), 5’-CACCGCTTGAGTTGGCTGGCTCGGG-3’; (2), 5’-CACCGTAACTTCACGAAGTTATCG-3’; Monkey SH3KBP1 gRNA: (1), 5’-GGCACAGAAUGAUGAUGAATT-3’; (2), 5’-CACCGATGAGCTGACGATCAGCGT-3’.

### Preparation of cytoplasmic and nuclear proteins

NE-PER nuclear and cytoplasmic extraction kit was used for testing according to the manufacturer’s instructions. Briefly, cells were harvested with ice-cold PBS and lysed by douncing 30 times in 500 μL membrane lysis buffer containing protease inhibitors. The homogenate was centrifuged at 500 g for 10 min. The supernatant was saved as cytosol, and the pellet was saved as crude nuclei. The crude nuclei were washed twice with 500 μL membrane lysis buffer and resuspended in 20–50 μL of extract buffer and shaken vigorously every 30 s for 15 min, followed by centrifugation at 15,000 *g* for 10 min. The supernatants containing nuclear proteins were saved for subsequent analysis.

### Statistical analysis

All the statistical analyses were performed with GraphPad Prism 8.4 software. Data are presented as the mean ± SD from three independent samples. Statistically significant differences between groups were determined using the Student’s *t*-test. Differences in data were considered statistically significant when the *P* value was less than 0.05.

## Supporting information

S1 Fig SH3KBP1 suppresses PRRSV replication. **(A)** Marc-145 cells were transfected with Myc-SH3KBP1 or Myc-EV for 24 h prior to infection with PRRSV (MOI = 0.1) and 36 h post-infection (hpi), the cell monolayer was fixed and stained for PRRSV N protein (green) and nuclei (blue) for evaluation by IFA. Scale bars, 200 μm. **(B)** Marc-145 cells were transfected with siSH3KBP1 or NC for 36 h prior to infection with PRRSV (MOI = 0.1) and were fixed for immunofluorescent staining of PRRSV (green). Scale bars, 200 μm. **(C-D)** CCK-8 assays were performed on Marc-145 cells that were transfected with either SH3KBP1 overexpression plasmids or siSH3KBP1.
(TIFF)

S2 Fig SH3KBP1 promotes IFN-β production and ISGs expression. **(A)** Western blot analysis of IFN-β, ISG15 and RANTES production in HEK-293T cells transfected with SH3KBP1 after infection with SeV. **(B)** ELISA analysis of RANTES production in the supernatants of HEK-293T cells transfected with SH3KBP1 after infection with SeV. **(C)** Western blot analysis of IFN-β, ISG15 and RANTES production in WT and SH3KBP1-KO HEK-293T cells after infection with SeV. **(D)** ELISA analysis of RANTES production in the supernatants of WT and SH3KBP1-KO HEK-293T cells after infection with SeV. Data are representative of three independent experiments. Data are expressed as mean ± SD replicates of three independent experiments (*P < 0.05, **P < 0.01, ***P < 0.001; unpaired, two-tailed Student’s *t* test).
(TIFF)

S3 Fig SH3KBP1 upregulates the protein level of RIG-I. **(A)** iPAM cells were transfected with the Myc-SH3KBP1 or Myc-EV, followed by Western blot with the indicated antibodies. **(B)** Marc-145 cells were transfected with Myc-SH3KBP1 or control plasmid, and the expression of RIG-I was detected by RT-qPCR. **(C)** Cell activity of WT and SH3KBP1-KO HEK-293T cells was detected by CCK-8 assay. The activity of SH3KBP1-KO cells was normalized to that of WT cells. Data are expressed as mean ± SD replicates of three independent experiments (*P < 0.05, **P < 0.01, ***P < 0.001; unpaired, two-tailed Student’s *t* test).
(TIFF)

S4 Fig TRIM25 deficiency attenuates the SH3KBP1-induced expression of IFN-β and ISGs. **(A)** HEK-293T cells were transfected with Flag-EV or Flag-RIG-I for 24 h. Co-IP and Western blot analyses were performed with indicated antibodies. **(B)** Cell activity of WT HEK-293 cells and TRIM25-KO cells was detected by CCK-8 assay. The activity of TRIM25-KO cells was normalized to that of WT cells. (**C)** Western blot analysis of IFN-β, ISG15 and RANTES production in WT and TRIM25-KO HEK-293 cells transfected with Myc-SH3KBP1 after infection with SeV. **(D)** ELISA analysis of RANTES production in the supernatants of WT and TRIM25-KO HEK-293 cells transfected with Myc-SH3KBP1 after infection with SeV. **(E)** Schematic of full-length SH3KBP1 and its truncation. HEK-293T cells were transfected with Myc-SH3KBP1 full length or truncations for 24 h, followed by Co-IP and Western blot analysis with TRIM25 antibody. **(F)** Schematic of full-length TRIM25 and its truncation. HEK-293T cells were transfected with HA-TRIM25 full length or truncations for 24 h, followed by Co-IP and Western blot analysis with indicated antibodies. Data are expressed as mean ± SD replicates of three independent experiments (*P < 0.05, **P < 0.01, ***P < 0.001; unpaired, two-tailed Student’s *t* test).
(TIFF)

S5 Fig Baf-A1 suppresses PRRSV replication but does not affect the adsorption and internalization of PRRSV. **(A)** PAM cells were infected with PRRSV (MOI = 0.1, 0.5, 1) for 36 h. The cell lysates were collected and the expression level of SH3KBP1 was detected by RT-qPCR. **(B)** RT-qPCR analysis of SH3KBP1 mRNA levels in HEK-293T cells transfected with Flag-EV or Flag-NSP2. **(C)** The expression levels of LC3 and SH3KBP1 in Marc-145 cells after PRRSV infection were detected by Western blot. **(D)** Adsorption assay. Cells were incubated with a mixture of Baf-A1/DMSO and PRRSV at 4°C for 1 h and then harvested for RT-qPCR. **(E)** Internalization assay. Cells were incubated with PRRSV (MOI = 0.1) at 4°C for 1 h, washed with PBS, and finally incubated with Baf-A1/DMSO for another 1 h at 37°C. The levels of PRRSV ORF7 mRNA were detected by RT-qPCR. **(F)** Replication assay. Cells were incubated with PRRSV (MOI = 0.1) for 24 h with Baf-A1/DMSO and then harvested for RT-qPCR. **(G)** HEK-293T cells were infected with SeV for 4, 8, and 12 h. The cell lysates were collected and the protein level of SH3KBP1 was detected by Western blot. **(H-J)** HEK-293T cells were treated with Baf-A1, MG132, Z-VAD and DMSO, inoculated with SeV, and samples were collected at 8 h, followed by Western blot with the indicated antibodies. The data are representative of results from three independent experiments. Data are expressed as mean ± SD replicates of three independent experiments (*P < 0.05, **P < 0.01, ***P < 0.001; unpaired, two-tailed Student’s *t* test).
(TIFF)

S6 Fig NSP2 interacts and colocalizes with SH3KBP1 **(A)** Marc-145 cells were transfected with plasmids expressing different PRRSV NSPs or Flag-EV. The level of SH3KBP1 protein was detected by Western blot after transfection. **(B)** iPAM cells were transfected with Flag-NSP2 or Flag-EV, and WCL collected at 24 h were subjected to IP with Flag antibodies. **(C)** WT and ATG5-KO cells were transfected with plasmids encoding Flag-NSP2, and then collected at 24 h for Western blot. **(D)** HEK-293T cells were transfected with Flag-NSP2-muants and Myc-SH3KBP1 for 24 h. Cells were immunostained with anti-Myc and Flag antibodies, observed under a confocal microscope. Scale bars, 10 μm. The colocalization coefficient was analyzed by image J.
(TIFF)

S1 Data Data that underlies this paper. Excel spreadsheet containing, in separate sheets, the underlying numerical data for Figs 1B–1D, 1F, 1G, 2A–2H, 3B–3G, 6G, 10C, 10E, 10F, S1C, S1D, S2B, S2D, S3B, S3C, S4B, S4D, S5A, S5B, S5D–S5F and S6D.
(XLSX)
