# Supplementary figures and images for: Porcine reproductive and respiratory syndrome virus nonstructural protein 2 promotes the autophagic degradation of adaptor protein SH3KBP1 to antagonize host innate immune responses by enhancing K63-linked polyubiquitination of RIG-I
Source: PLoS Pathog. 2024 Oct 28;20(10):e1012670. doi: 10.1371/journal.ppat.1012670 (PMC11560026; doi:10.1371/journal.ppat.1012670)

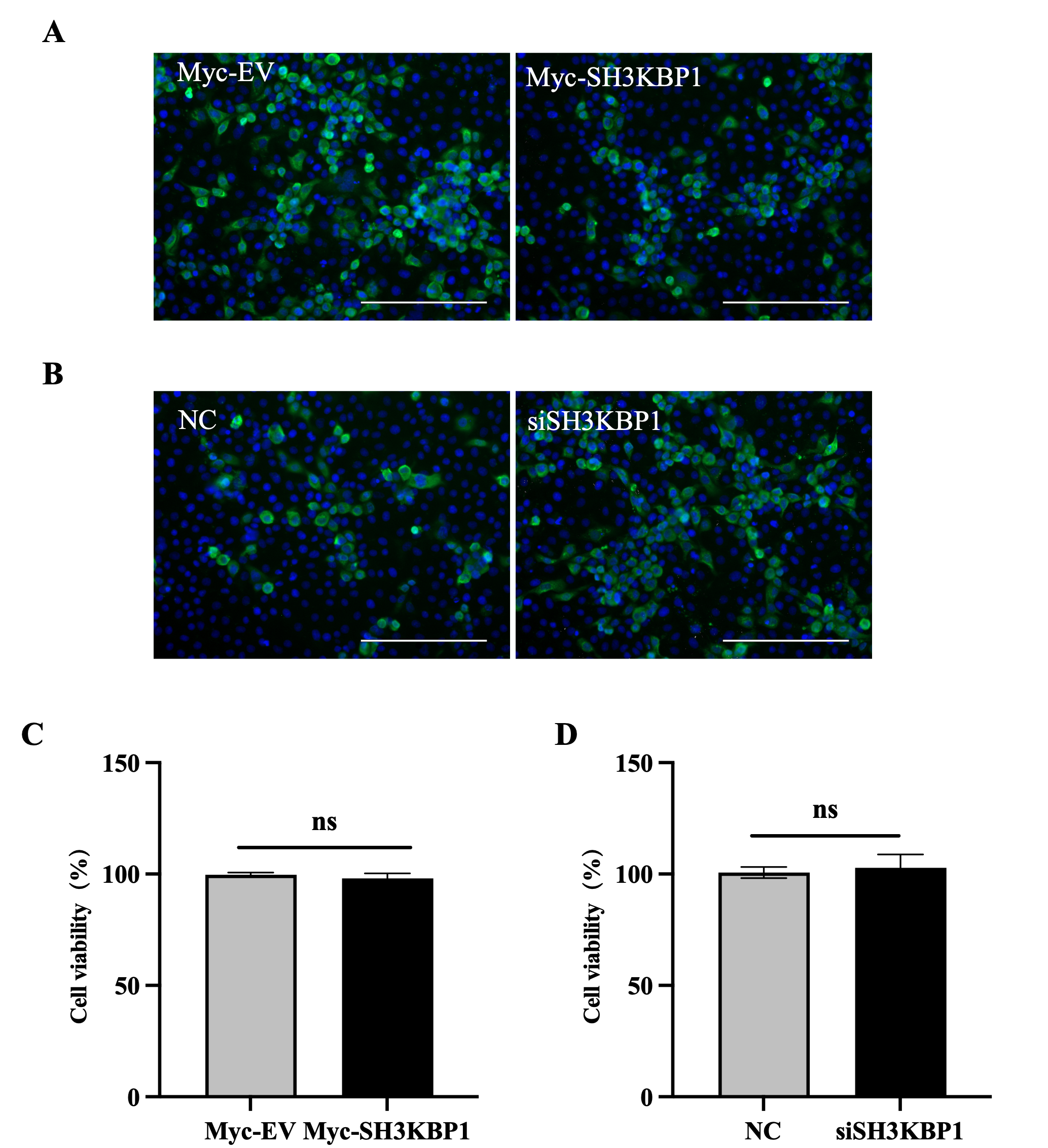

Supplement: S1 Fig — (A) Marc-145 cells were transfected with Myc-SH3KBP1 or Myc-EV for 24 h prior to infection with PRRSV (MOI = 0.1) and 36 h post-infection (hpi), the cell monolayer was fixed and stained for PRRSV N protein (green) and nuclei (blue) for evaluation by IFA. Scale bars, 200 μm. (B) Marc-145 cells were transfected with siSH3KBP1 or NC for 36 h prior to infection with PRRSV (MOI = 0.1) and were fixed for immunofluorescent staining of PRRSV (green). Scale bars, 200 μm. (C-D) CCK-8 assays were performed on Marc-145 cells that were transfected with either SH3KBP1 overexpression plasmids or siSH3KBP1. (TIFF) [file ppat.1012670.s001.tiff]

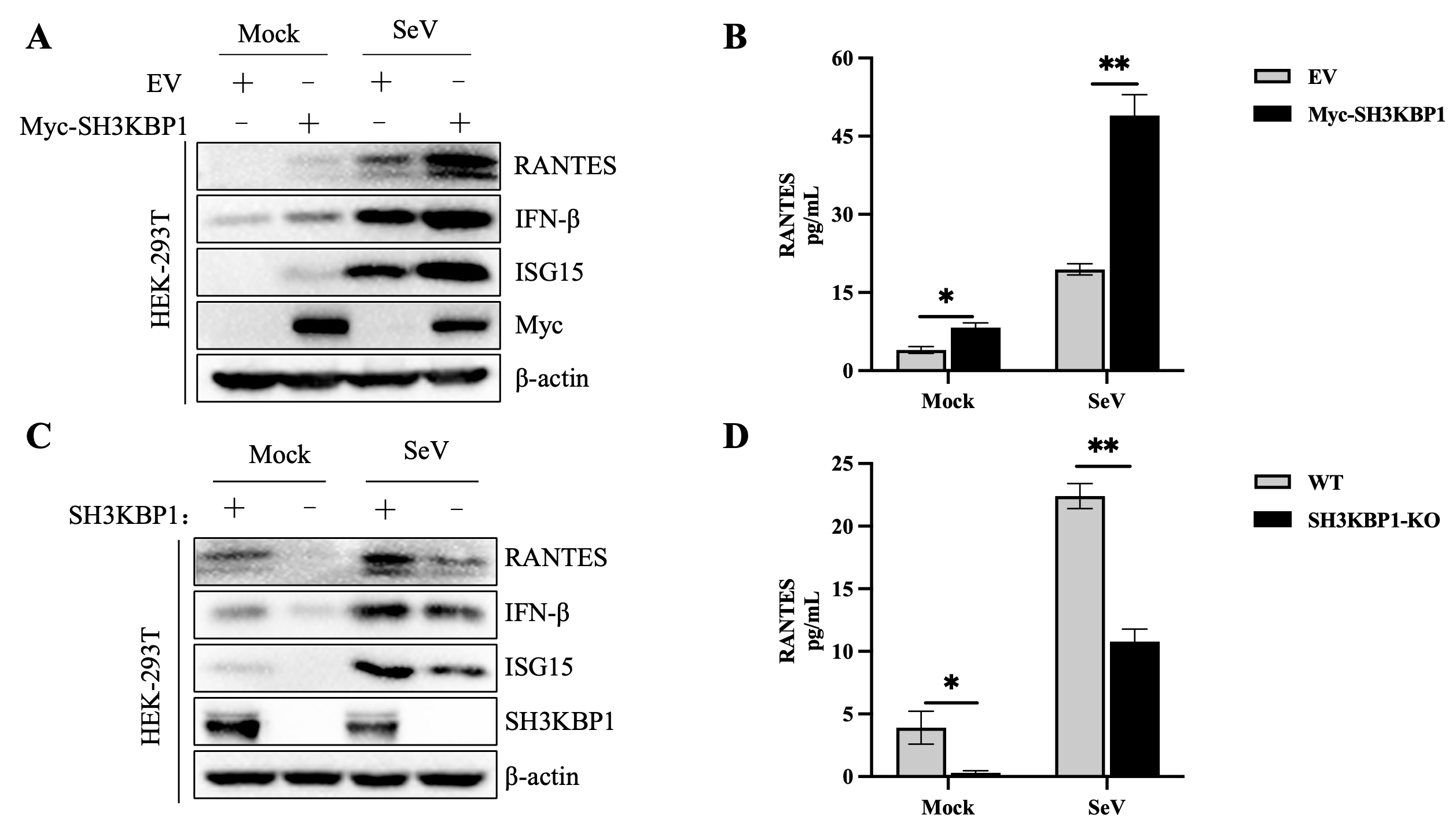

Supplement: S2 Fig — (A) Western blot analysis of IFN-β, ISG15 and RANTES production in HEK-293T cells transfected with SH3KBP1 after infection with SeV. (B) ELISA analysis of RANTES production in the supernatants of HEK-293T cells transfected with SH3KBP1 after infection with SeV. (C) Western blot analysis of IFN-β, ISG15 and RANTES production in WT and SH3KBP1-KO HEK-293T cells after infection with SeV. (D) ELISA analysis of RANTES production in the supernatants of WT and SH3KBP1-KO HEK-293T cells after infection with SeV. Data are representative of three independent experiments. Data are expressed as mean ± SD replicates of three independent experiments (*P < 0.05, **P < 0.01, ***P < 0.001; unpaired, two-tailed Student’s t test). (TIFF) [file ppat.1012670.s002.tiff]

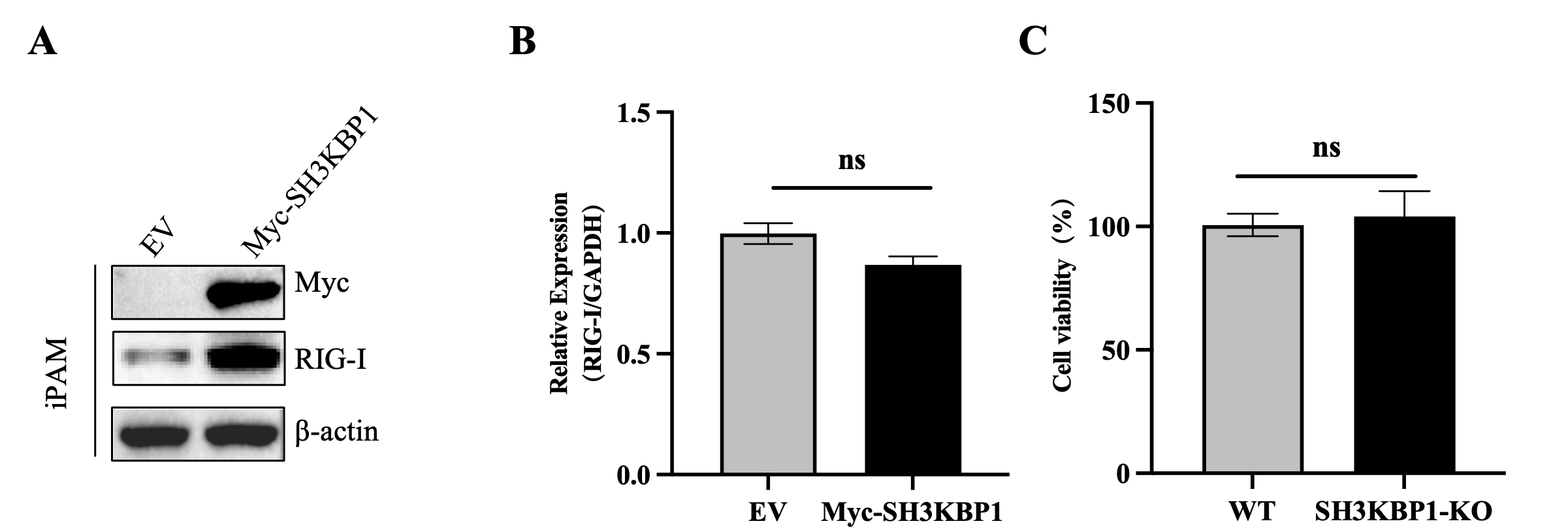

Supplement: S3 Fig — (A) iPAM cells were transfected with the Myc-SH3KBP1 or Myc-EV, followed by Western blot with the indicated antibodies. (B) Marc-145 cells were transfected with Myc-SH3KBP1 or control plasmid, and the expression of RIG-I was detected by RT-qPCR. (C) Cell activity of WT and SH3KBP1-KO HEK-293T cells was detected by CCK-8 assay. The activity of SH3KBP1-KO cells was normalized to that of WT cells. Data are expressed as mean ± SD replicates of three independent experiments (*P < 0.05, **P < 0.01, ***P < 0.001; unpaired, two-tailed Student’s t test). (TIFF) [file ppat.1012670.s003.tiff]

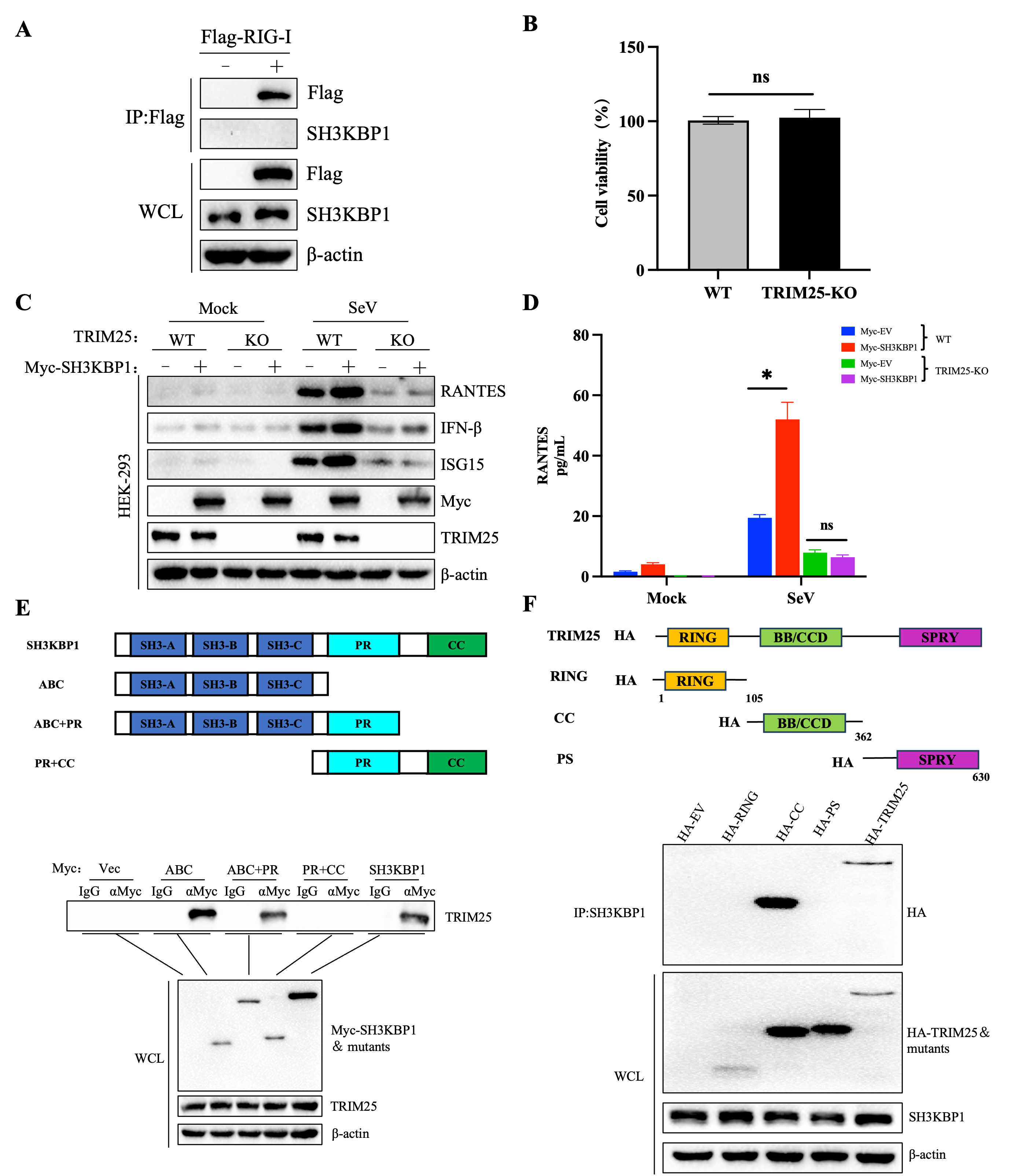

Supplement: S4 Fig — (A) HEK-293T cells were transfected with Flag-EV or Flag-RIG-I for 24 h. Co-IP and Western blot analyses were performed with indicated antibodies. (B) Cell activity of WT HEK-293 cells and TRIM25-KO cells was detected by CCK-8 assay. The activity of TRIM25-KO cells was normalized to that of WT cells. (C) Western blot analysis of IFN-β, ISG15 and RANTES production in WT and TRIM25-KO HEK-293 cells transfected with Myc-SH3KBP1 after infection with SeV. (D) ELISA analysis of RANTES production in the supernatants of WT and TRIM25-KO HEK-293 cells transfected with Myc-SH3KBP1 after infection with SeV. (E) Schematic of full-length SH3KBP1 and its truncation. HEK-293T cells were transfected with Myc-SH3KBP1 full length or truncations for 24 h, followed by Co-IP and Western blot analysis with TRIM25 antibody. (F) Schematic of full-length TRIM25 and its truncation. HEK-293T cells were transfected with HA-TRIM25 full length or truncations for 24 h, followed by Co-IP and Western blot analysis with indicated antibodies. Data are expressed as mean ± SD replicates of three independent experiments (*P < 0.05, **P < 0.01, ***P < 0.001; unpaired, two-tailed Student’s t test). (TIFF) [file ppat.1012670.s004.tiff]

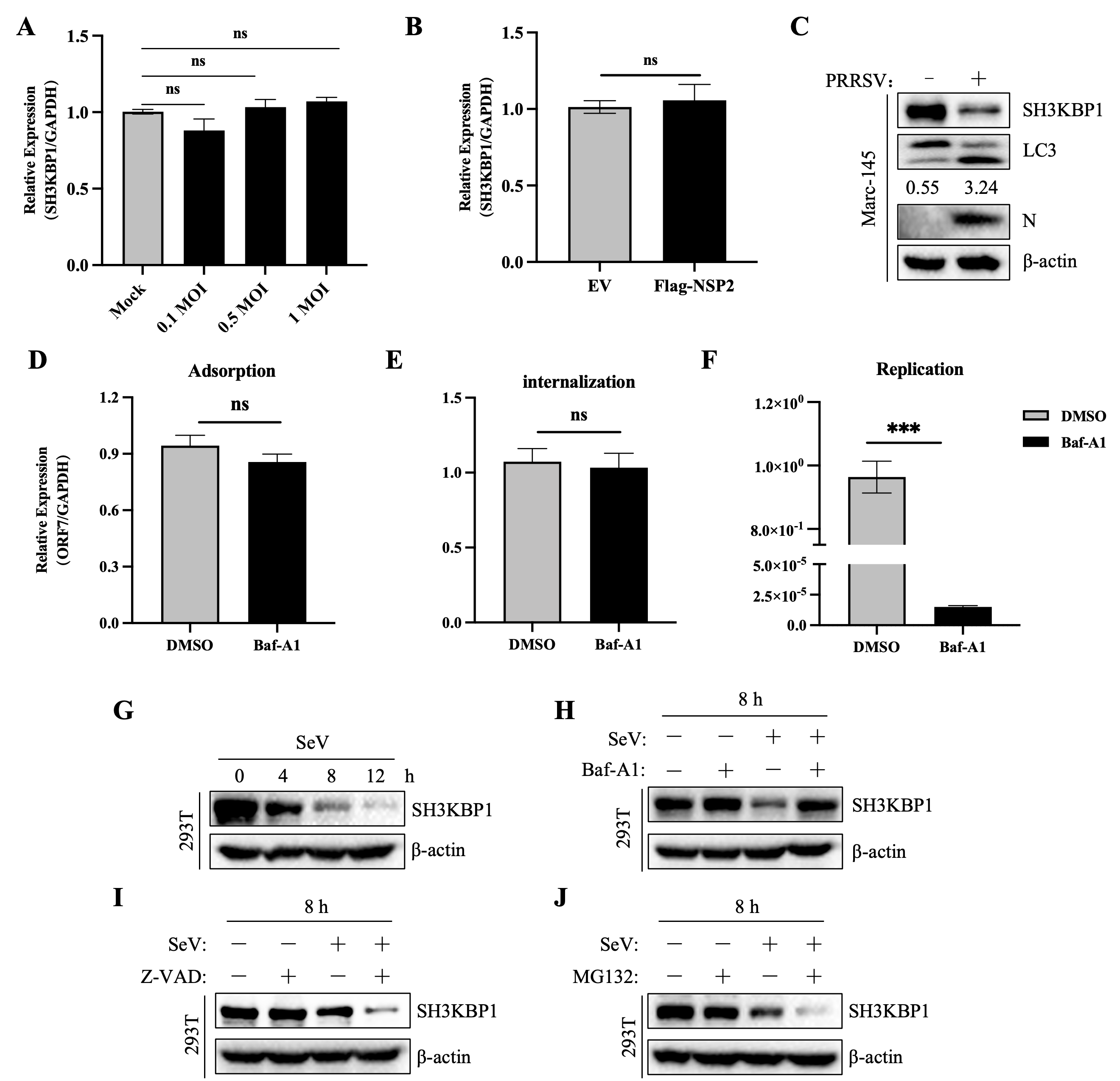

Supplement: S5 Fig — (A) PAM cells were infected with PRRSV (MOI = 0.1, 0.5, 1) for 36 h. The cell lysates were collected and the expression level of SH3KBP1 was detected by RT-qPCR. (B) RT-qPCR analysis of SH3KBP1 mRNA levels in HEK-293T cells transfected with Flag-EV or Flag-NSP2. (C) The expression levels of LC3 and SH3KBP1 in Marc-145 cells after PRRSV infection were detected by Western blot. (D) Adsorption assay. Cells were incubated with a mixture of Baf-A1/DMSO and PRRSV at 4°C for 1 h and then harvested for RT-qPCR. (E) Internalization assay. Cells were incubated with PRRSV (MOI = 0.1) at 4°C for 1 h, washed with PBS, and finally incubated with Baf-A1/DMSO for another 1 h at 37°C. The levels of PRRSV ORF7 mRNA were detected by RT-qPCR. (F) Replication assay. Cells were incubated with PRRSV (MOI = 0.1) for 24 h with Baf-A1/DMSO and then harvested for RT-qPCR. (G) HEK-293T cells were infected with SeV for 4, 8, and 12 h. The cell lysates were collected and the protein level of SH3KBP1 was detected by Western blot. (H-J) HEK-293T cells were treated with Baf-A1, MG132, Z-VAD and DMSO, inoculated with SeV, and samples were collected at 8 h, followed by Western blot with the indicated antibodies. The data are representative of results from three independent experiments. Data are expressed as mean ± SD replicates of three independent experiments (*P < 0.05, **P < 0.01, ***P < 0.001; unpaired, two-tailed Student’s t test). (TIFF) [file ppat.1012670.s005.tiff]

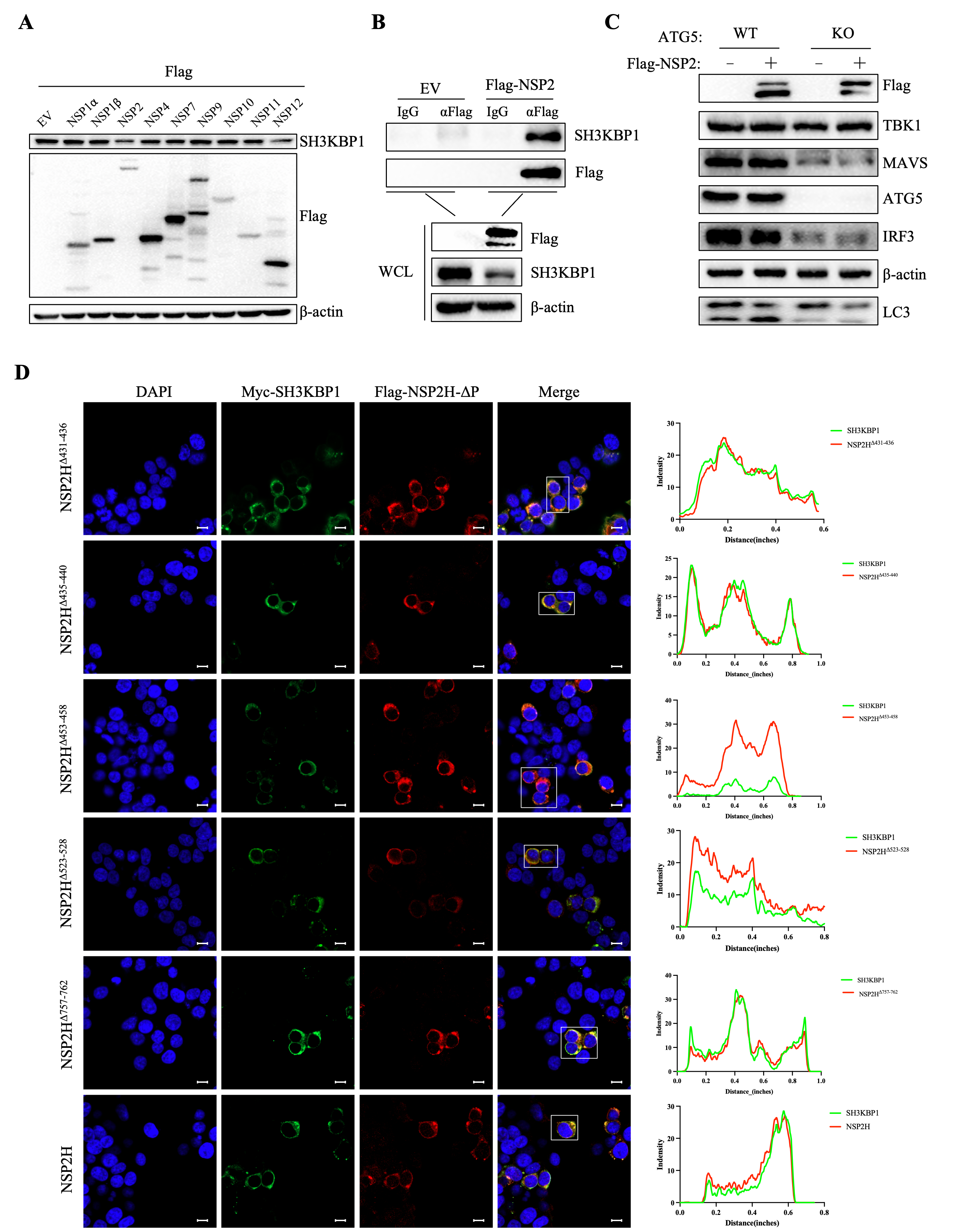

Supplement: S6 Fig — NSP2 interacts and colocalizes with SH3KBP1 (A) Marc-145 cells were transfected with plasmids expressing different PRRSV NSPs or Flag-EV. The level of SH3KBP1 protein was detected by Western blot after transfection. (B) iPAM cells were transfected with Flag-NSP2 or Flag-EV, and WCL collected at 24 h were subjected to IP with Flag antibodies. (C) WT and ATG5-KO cells were transfected with plasmids encoding Flag-NSP2, and then collected at 24 h for Western blot. (D) HEK-293T cells were transfected with Flag-NSP2-muants and Myc-SH3KBP1 for 24 h. Cells were immunostained with anti-Myc and Flag antibodies, observed under a confocal microscope. Scale bars, 10 μm. The colocalization coefficient was analyzed by image J. (TIFF) [file ppat.1012670.s006.tiff]
